# Evidence for lactone formation during infrared multiple photon dissociation spectroscopy of bromoalkanoate doped salt clusters†

**DOI:** 10.1039/d0cp00272k

**Published:** 2020-06-04

**Authors:** Nina K. Bersenkowitsch, Milan Ončák, Jakob Heller, Tobias F. Pascher, Christian van der Linde, Martin K. Beyer

**Affiliations:** Institut für Ionenphysik und Angewandte Physik, Universitat Innsbruck, Technikerstraβe 25, 6020 Innsbruck, Austria

## Abstract

Reaction mechanisms of organic molecules in a salt environment are of fundamental interest and are potentially relevant for atmospheric chemistry, in particular sea-salt aerosols. Here, we found evidence for lactone formation upon infrared multiple photon dissociation (IRMPD) of non-covalent bromoalkanoate complexes as well as bromoalkanoate embedded in sodium iodide clusters. The mechanism of lactone formation from bromoalkanoates of different chain lengths is studied in the gas phase with and without salt environment by a combination of IRMPD and quantum chemical calculations. IRMPD spectra are recorded in the 833-3846 cmT^1^ range by irradiating the clusters with tunable laser systems while they are stored in the cell of a Fourier transform ion cyclotron resonance (FT-ICR) mass spectrometer. The measurements of the binary complex Br(CH2)_m_COOH·Br(CH2)_m_COO^-^ for *m* = 4 indicate valerolactone formation without salt environment while lactone formation is hindered for longer chain lengths. When embedded in sodium iodide clusters, butyrolactone formation from 4-bromobutyrate seems to take place already during formation of the doped clusters in the electrospray process, evidenced by the infrared (IR) signature of the lactone. In contrast, IRMPD spectra of sodium iodide clusters containing 5-bromovalerate contain signatures for both valerate as well as valerolactone. In both cases, however, a neutral fragment corresponding to the mass of valerolactone is eliminated, indicating that ring formation can be activated by IR light in the salt cluster. Quantum chemical calculations show that already complexation with one sodium ion significantly increases the barrier for lactone formation for all chain lengths. IRMPD of sodium iodide clusters doped with neutral bromoalkanoic acid molecules proceeds by elimination of HI or desorption of the intact acid molecule from the cluster.

## Introduction

When it comes to modelling the climate, the investigation of aerosols plays an important role.^1–5^ Tropospheric aerosols contribute to processes such as cloud formation.^6^ As the ocean covers more than 70% of the Earths’ surface, marine aerosols^7^ constitute a significant fraction of the tropospheric aerosol inventory. They are produced *via* the mechanical disruption of the sea surface.^8^ Marine aerosols are composed of a complex mixture of species that include not only salt, but also a large amount of organic matter.^9–12^ The exposure to intense solar radiation induces complex photoprocessing of organic matter in sea-salt aerosols.^13,14^ It is known that carbonaceous particles with acidic character appear with a quite high concentration in marine aerosols.^15,16^ Volatile organic compounds^17^ (VOCs) from anthropogenic and biogenic sources undergo rapid transformation *via* photolysis.^18^

Halogens are very reactive species, and brominated alkanes are reported to be a source of bromine in the stratosphere^19^ where bromine participates in the catalytic destruction of ozone.^20^ Several studies reported the presence of *n*-alkanes and *n*-alkanoic acids in aerosols.^21–24^ Ground water pollution^25–29^ is a highly relevant topic as it affects directly human health^27,30,31^ and environment.^32^ Inorganic^33,34^ and organic^35–38^ pollutants as some (di-)bromoalkanoic pesticides^39–41^ and volatile halogenated bromoalkanes^42^ have already been identified. 3-Bromopyruvic acid is a potential anti-cancer drug and, if applied to patients, will make its way *via* wastewater to the oceans.^43^ Vogel *et al.* ^44^ studied the reactivity of some mono- and di-bromoalkanes in aqueous buffers. A large variety of halogenated organic molecules are formed as disinfection byproducts in the reaction of disinfectants with natural organic matter and bromide or iodide.^45^ These pollutants can easily get into the ocean, and can be therefore present in sea salt aerosols.

Visible or near-infrared one photon vibrational overtone excitation has the potential to trigger atmospheric reactions.^46^ In laboratory experiments, however, infrared multiple photon dissociation (IRMPD) is usually performed at longer wavelengths to obtain action spectra of clusters and ions.^47–50^ It is well established that resonant infrared irradiation may activate chemical reactions in clusters, used *e.g.* to characterize ion-molecule reaction products.^51,52^ A detailed IRMPD study of N_2_O decomposition on rhodium clusters by Mackenzie and co-workers revealed the influence of co-adsorbed oxygen.^53^ However, even black-body infrared radiation is able to trigger N_2_O decomposition on small rhodium clusters, showing that no mode-specificity is involved.^54^ We have recently investigated the IR induced decomposition of copper formate,^55,56^ and measured IRMPD spectra of CO_2_^∙–^(H_2_O)_*n*_ and Co(CO_2_)(H_2_O)_*n*_^−^.^57,58^ Of particular interest to the present work, however, are our recent studies on salt clusters doped with organic molecules, where we used IRMPD to elucidate structural properties^59,60^ and UV excitation to analyze the influence of the salt environment on glyoxylate photochemistry.^61^

To get a better understanding of the chemistry of brominated organic molecules in a salt environment, we performed IRMPD on anionic complexes of *n*-bromoalkanoic acid (*n* = 5, 8) and the corresponding *n*-bromoalkanoate, *i.e.* Br(CH_2_)_*n*–1_COOH·Br(CH_2_)_*n*–1_COO^−^. We then repeated the experiments with sodium iodide clusters in which one I^−^ ion was replaced by a bromoalkanoate ion, Na_6_I_4_Br(CH_2_)_*n*–1_COO^+^, as well as sodium iodide cluster with one molecule of bromoalkanoic acid adsorbed, Na_6_I_5_Br(CH_2_)_*n*–1_COOH^+^. Elimination of neutral (CH_2_)_*n*–1_OCO, which indicates lactone formation, is exclusively observed for bromoalkanoate species. Complexation with salt reduces the propensity for (CH_2_)_*n*–1_OCO elimination. Quantum chemical calculations provide a molecular level understanding of the observed IRMPD pathways.

## Experimental and computational details

The detailed setup of the experiment was already described elsewhere.^62^ Briefly, the main setup consists of a Bruker APEX Qe 9.4 T Fourier-Transform Ion Cyclotron Resonance (FT-ICR) Mass Spectrometer, equipped with an ESI/MALDI Dual Source II and a Nanobay Console. Doped sodium iodide clusters are generated by electrospray ionization (ESI) of a 5 mM NaI solution in a 1:1 mixture of MeOH: H_2_O, containing the respective bromoalkanoic acid at a concentration of 1–5 mM. All chemicals were purchased from Sigma-Aldrich, with a purity of at least 98%. Mass selected cluster ions are irradiated with light from two tunable laser systems, emitting IR light at 3846–2234 cm^−1^ (EKSPLA NT277, 1000 Hz repetition rate, typical power 25–100 mW) and at 2234-833 cm^−1^ (EKSPLA NT273-XIR, 1000 Hz repetition rate, typical power 2–30 mW). A plot of the laser power as a function of wavelength is available as ESI,† Fig. S1. IR absorption cross sections are derived by assuming sequential photon absorption following first-order kinetics, as described in detail before,^59^ not considering radiative cooling. The contribution of ambient black-body infrared radiative dissociation (BIRD)^63^ was taken into account as much as possible. For single photon cross sections, the rate of unimolecular decomposition due to BIRD^59^ was measured by storing the ions for different time intervals without laser irradiation. For multiphoton cross sections, the fragment intensity due to BIRD was subtracted before further analysis. IRMPD spectra are recorded by scanning the laser frequency and monitoring the intensity of the dissociation products by mass spectrometry. The influence of the laser pulse energy is corrected within the calculation of the displayed cross sections and IRMPD yield. Overview spectra were obtained in the region where absorptions could be expected by scanning the laser typically in steps of 20–30 nm. The absorption bands identified in this way were re-measured with smaller steps and are shown here. Overview spectra are available in the ESI† (Fig. S2 and S3).

The clusters were investigated using methods of computational chemistry at the B3LYP/def2TZVP level of theory, considering several isomers for each ion. For the bromoalkanoic acid/alkanoate complex, different conformations of the COOH hydrogen bond were considered, with and without lactone formation. For salt complexes, we employed different Na_6_I_5_ clusters^59^ and replaced one I^−^ ion by the respective alkanoate. Calculated IR spectra were scaled by a factor of 0.97 and displayed with Gaussian broadening with a full width at half maximum (FWHM) of 30 cm^−1^. All structures represent local minima on the potential energy surface. Relative energies are reported with zero-point energy correction, but without thermal corrections. All calculations were performed in the Gaussian program.^64^

## Results and discussion

### Evidence for lactone formation in Br(CH_2_)_m_COOH·Br(CH_2_)_m_COO^−^ (*m* = 4, 7)

**Fig. 1:**
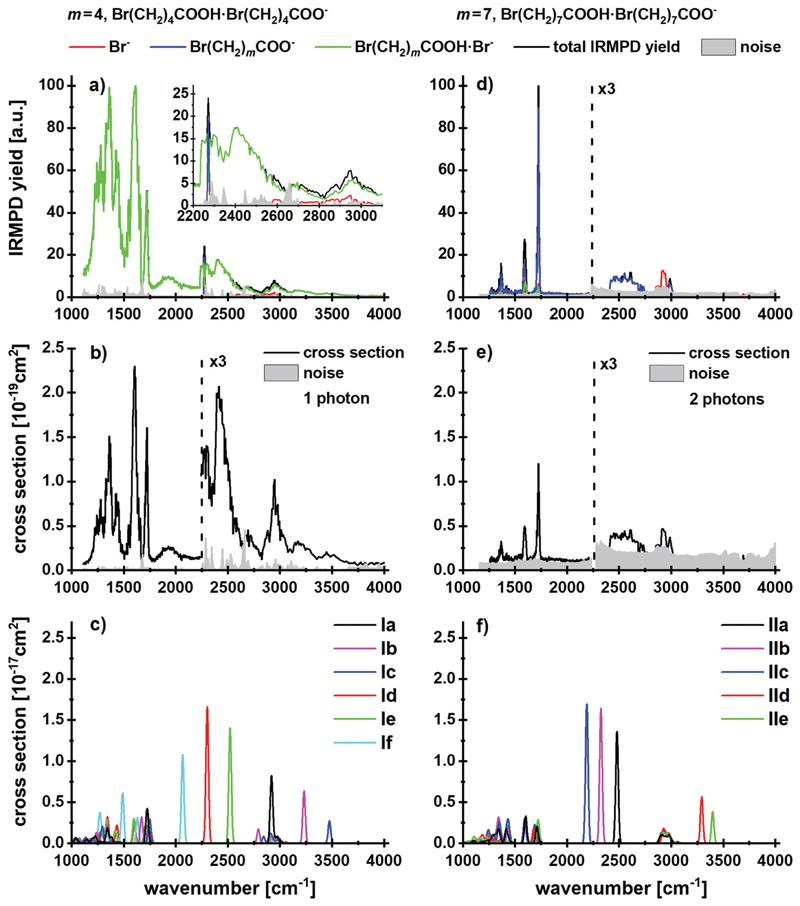
(a and d) IRMPD yield spectra with the total yield (black line) and the corresponding fragment contributions (red, blue and green lines), (b and e) experimental and (c and f) theoretical cross sections of the cluster Br(CH2)_m_COO·Br(CH_2_)_m_COOH^−^ with *m* = 4 and *m* = 7. In (d), the Br^−^ and Br(CH_2_)7COOH·Br^−^ fragments are superimposed at 1500–1750 cm^−1^. Calculated at the B3LYP/def2TZVP level of theory.

**Fig. 2:**
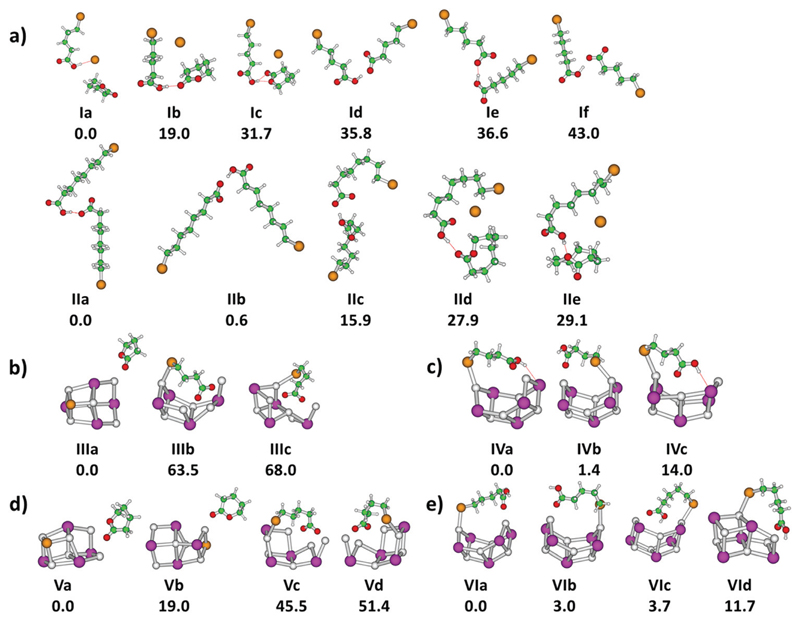
(a) *n*-Bromoalkanoic acid-alkanoate pairs; (b) 4-bromobutyrate/Na_6_I_4_; (c) 4-bromobutyric acid/Na_6_I_5_; (d) 5-bromovalerate/Na_6_I_4_; (e) 5-bromo-valeric acid/Na_6_I_5_. Calculated at the B3LYP/def2TZVP level of theory, relative energy is given in kJ mol^−1^. Color code: Na grey, I violet, Br brown, C green, O red, H white.

First, we focus on the anionic complexes Br(CH_2_)_m_COOH·Br(CH_2_)_m_COO^−^ to identify potential reaction pathways of bromoalkanoic acids and bromoalkanoates in the gas phase, in the absence of a salt environment. Fig. 1a and d shows the IRMPD yield spectra normalized to the most intense absorption, and Fig. 1b and e illustrates the experimental multiphoton cross sections of these species for *m* = 4, 7. Calculated structures of selected low-lying isomers are shown in Fig. 2 with the corresponding spectra in Fig. 1c and f. Two structure types are considered, with and without pre-formed lactone. For *m* = 4, the more stable isomers **Ia–c** contain lactone while isomers **Id–f** feature the intact alkanoate Br(CH_2_)_4_COO^−^. For *m* = 7, the structures containing lactone, isomers **IId-e**, lie higher in energy than the alkanoate. Table 1 summarizes the reactions, energetics and branching ratios of the fragments, where the latter were calculated *via* the integrated experimental curves. The notations IR1, IR2 and IR3 indicate different infrared regions, the respective wavenumber range is noted in the table. Note that for *m* = 3, the Br(CH_2_)_3_COOH·Br(CH_2_)_3_COO^−^ precursor could not be obtained in the experiment; the spectrum for *m* = 10 is shown in the ESI,† Fig. S4 and S5. The kinetics for *m* = 4, 7, 10 are shown in Fig. S6-S8 (ESI†).

**Scheme 1:**
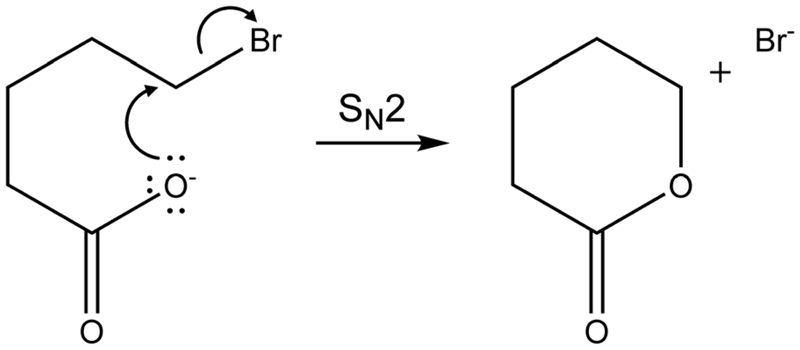
Formation of valerolactone *via* an intramolecular S_N_2 reaction from Br(CH_2_)_4_COO^−^.

Three different fragments are observed: Br(CH_2_)_m_COO^−^ is obviously formed by loss of neutral Br(CH_2_)_m_COOH, while Br^−^ and Br(CH_2_)_m_COOH·Br^−^ suggest lactone formation upon IRMPD, see Table 1. For *m* = 4, the by far dominant IRMPD fragment is Br(CH_2_)_4_COOH·Br^−^, the eliminated (CH_2_)_4_OCO is most likely valerolactone. Scheme 1 depicts the mechanism of the S_N_2 reaction that transforms Br(CH_2_)_4_COO^−^ to (CH_2_)_4_OCO + Br^−^. Although the alternative reaction, decarboxylation and (CH_2_)_4_ cyclobutane formation is thermochemically slightly favored with a reaction energy of Δ*E =* 34 kJ mol^−1^, calculations indicate a high barrier of 168 kJ mol^−1^ in Br(CH_2_)_4_COO^−^ due to an energetically demanding charge transfer. Valerolactone is therefore the most plausible product. The bromide fragment appears selectively around 2600–3000 cm^−1^ where high photon energies and high laser power provide more energy to trigger secondary reactions, in this case lactone formation followed by dissociation of the non-covalent Br(CH_2_)_4_COOH·Br^−^ complex. In line with experiment, the calculated reaction energies in Table 1 predict formation of Br(CH_2_)_4_COOH·Br^−^ to be the most probable channel; evaporation of the pre-formed lactone requires only 42 kJ mol^−1^. Other channels lie considerably higher in energy.

The main fragment observed for *m* = 7 is Br(CH_2_)_7_COO^−^ with evaporation of Br(CH_2_)_7_COOH, while Br^−^ and Br(CH_2_)_7_COOH·Br^−^ are present only in minor amounts. Br^−^ is observed again selectively around 2900 cm^−1^, while both Br^−^ and Br(CH_2_)_7_COOH·Br^−^ appear almost with the same yield at 1500–1750 cm^−1^. The calculated energies, Table 1, indicate that lactone is not present in major amounts on the cluster, as it would be eliminated preferentially, requiring 34 kJ mol^−1^, followed by Br^−^ production as the second most important channel. Instead, Br(CH_2_)_7_COO^−^ is formed from Br(CH_2_)_7_COOH·Br(CH_2_)_7_COO^−^in the experiment, with a dissociation energy of 76 kJ mol^−1^.

Analysis of the IR spectra supports the conclusions drawn on a thermochemical basis. The calculated isomers can be discriminated based on the position of the O–H stretch vibration. For *m* = 4, the broad absorption at 2200–2600 cm^−1^ is assigned to the O–H stretching mode shifted by the interaction with the COO^−^ group of the alkanoate in isomers **Id-f**. The tail of the spectrum at 3100–3600 cm^−1^, however, can be assigned to the interaction of the O–H group hydrogen bonded in the lactone complexes **Ia-c** and C-H stretching vibrations. The peak positions in the fingerprint region of 1100–1700 cm^−1^ support the presence of both **Ia-c** and **Id-f** isomer classes. However, no specific isomers can be assigned here. The peak centered at 1606 cm^−1^ is likely due to isomers **Id-f**, see also Fig. S5 (ESI†) for a zoom in the fingerprint region. The observed fragmentation can be induced by only one photon. The calculated and experimental cross sections, Fig. 1b and c, differ by one to two orders of magnitude, which we attribute in part to the high structural flexibility of the alkanoic acid/alkanoate-system, in particular the extreme broadening of the O–H stretching mode upon hydrogen bonding to the carboxylate group, which goes along with efficient energy redistribution. Radiative cooling may also contribute to the low experimental IRMPD yield.

In the IR spectrum for *m* = 7, the noise in the high-energy region above 3000 cm^−1^ is too high to identify or rule out the presence of octalactone based on the O–H vibrations. Besides the dominant evaporation of the intact acid, the fragments indicating lactone elimination, Br^−^ and Br(CH_2_)_7_COOH·Br^−^, are observed. All features in the spectrum can be assigned to isomer **IIa** without lactone. While there is an overlap with isomers containing lactone at the absorptions around 3000 cm^−1^ and 1700 cm^−1^, the calculated intense O–H stretch features of isomers **IId,e** at 3300–3400 cm^−1^ are missing in the experimental spectrum. Thus, the lactone containing isomers **IId,e** are at most a minor fraction of the experimental mixture. According to the calculated thermochemistry, at least two photons are required to induce the observed fragmentation; the experimental and calculated absorption cross sections differ again by two orders of magnitude.

### IRMPD of *n*-bromoalkanoic acids and *n*-bromoalkanoates in salt environment (*n* = 4, 5, 8, 11)

**Fig. 3:**
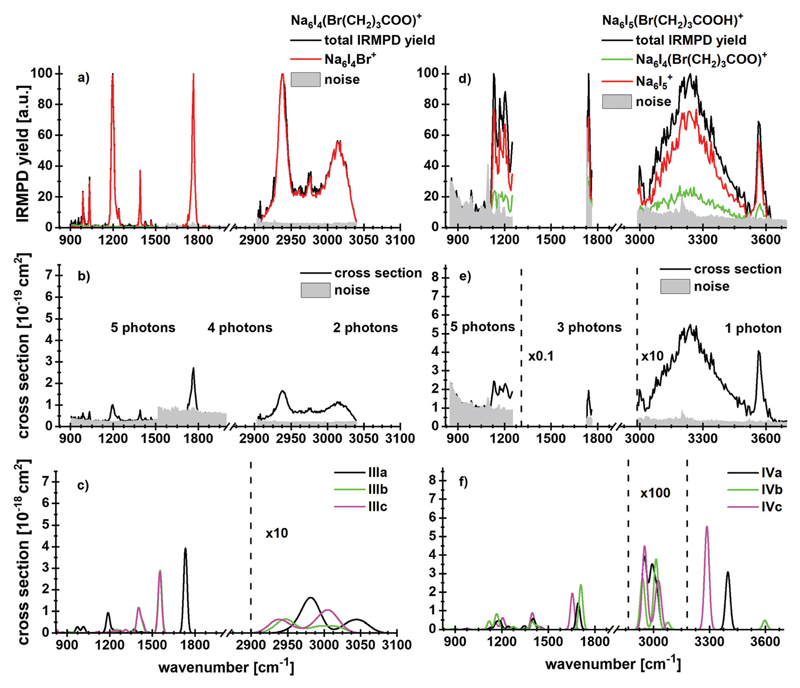
Experimental IRMPDyield spectra of (a) Na_6_I_4_(Br(CH_2_)_3_COO)^+^ and (d) Na_6_I_5_(Br(CH_2_)_3_COOH)^+^ with contribution of the most intense fragmentation products. (b and e) Multiphoton photodissociation cross sections derived from (a and d). (c and f) Calculated spectra in harmonic approximation for Na_6_I_4_(Br(CH_2_)_3_COO)^+^ and Na_6_I_5_(Br(CH_2_)_3_COOH)^+^, respectively, for 833–3846 cm^−1^. Here, the regions measured in detail are shown. The overview spectra are provided in Fig. S2 (ESI†). The theoretical spectra are scaled with a factor of 0.97.

To investigate the influence of a salt environment on the potential lactone formation, sodium iodide clusters were prepared by ESI, where one I^−^ ion was replaced by a bromoalk-anoate ion. Sodium iodide clusters doped with a neutral molecule of bromoalkanoic acid were studied for comparison. Table 1 shows the reaction energies and branching ratios of the fragments with an intensity above 4%, the detailed breakdown of all observed fragments is given in Table S1 (ESI†). Fig. 3 shows the absorption spectrum of Na_6_I_4_(Br(CH_2_)_3_COO) and Na_6_I_5_(Br(CH_2_)_3_COOH)^+^ in the 833–3846 cm^−1^ region. The spectra for Na_6_I_4_(Br(CH_2_)_3_COO) were measured with 15 s (region IR1), 7 s (region IR2) and 5 s (region IR3) irradiation time, while the ones for Na_6_I_5_(Br(CH_2_)_3_COOH)^+^ were measured with 15 s (region IR1), 6 s (region IR2) and 3 s (region IR3). The calculated structures of the most stable isomers are illu-strated in Fig. 2b-e. For the Na_6_I_4_(Br(CH_2_)_3_COO) ion, the most intense fragmentation channel over the whole spectral range is Na_6_I_4_Br, which indicates butyrolactone elimination from the cluster. In line with this interpretation, the most stable structure of Na_6_I_4_(Br(CH_2_)_3_COO) is Na_6_I_4_Br with (CH_2_)_3_OCO non-covalently attached, isomer **IIIa** in Fig. 2b. Since structures with a bromobutyrate moiety lie significantly higher in energy, isomer **IIIa** is also the most likely structure to appear in the experiment. The kinetics suggests that lactone elimination is a primary process (Fig. S10, ESI†).

The peaks at 2938 cm^−1^ and 3013 cm^−1^ do not allow for an unambiguous assignment of isomers, since C–H absorptions are only moderately influenced by the cluster structure. The observed features are consistent with any of the three calculated isomers, or a mixture of them. However, the sharp C–O stretch peak at 1761 cm^−1^, the missing absorption at 1540 cm^−1^ and the absorptions at 1195,1032 and 987 cm^−1^ are consistent with the dominant presence of isomer **IlIa**. This indicates that butyrolactone is formed in the electrospray process, before the clusters are stored in the ICR cell and heated by IR irradiation.

The dissociation into Na_6_I_4_Br^+^, observed as the main reaction channel in the experiment, requires 99 kJ mol^−1^, Table 1. This implies that about two photons are needed in the 2903–3040 cm^−1^ region and four to five photons in the fingerprint region. The absolute values of the experimental and theoretical cross section derived using this assumption (Fig. 3b and c) match well in the C–H stretch region, while the deviation is about an order of magnitude in the fingerprint region. Due to the low laser power in this region and the large number of degrees of freedom, irradiation times are long, which favors radiative cooling. This effect is not accounted for in our multiple photon analysis.

When neutral bromobutyric acid is attached to the sodium iodide cluster, resulting in Na_6_I_5_(Br(CH_2_)_3_COOH)^+^, the most intense fragmentation channel corresponds to evaporation of the intact 4-bromobutyric acid molecule from the cluster, evidenced by the detection of Na_6_I_5_ (see Fig. 3d-f for the spectra, Table 1 for the reaction energies and Fig. S11 (ESI†) for the kinetics). The second most intense channel is HI elimination. Evaporation of 4-bromobutyric acid or HI requires 99 kJ moP^1^ or 77 kJ mol^-1^, respectively. Following HI elimination, the remaining bromobutyrate affords lactone formation, which reduces the overall reaction energy to 14 kJ mol^−1^. However, both HI elimination and lactone formation are hindered by barriers (see also below). We note that HI release was already observed in a previous study where cesium iodide clusters were doped with small peptides.^60^ In both cases, HI elimination probably follows a similar mechanism as the release of HCl from a bulk sea-salt surface following uptake of HNO_3_, which was reported by Haan and Finlayson-Pitts.^65^

The IR spectrum shows that the cluster is present in several conformations. For example, in the O–H stretching region, the peak at 3569 cm^−1^ results from a free O–H vibration (*e.g.* in isomer **IVb**), whereas the broad absorption centered at 3241 cm^−1^ is induced by the OH… I^−^ interaction (isomers **IVa** and **IVc**). In the 2800–3000 cm^−1^ region, C–H vibrations contribute to the experimental spectral intensity. For the fragmentation in this wavelength region, at least one photon is needed. The peak at 1739 cm^−1^ points towards isomer **IVb** or similar conformations, with at least three photons needed for the dissociation. The experimental and theoretical cross section values are comparable in this region. Since the signal and the absorption around 1200 cm^−1^ were very weak, no assignment to a specific isomer is possible.

**Fig. 4:**
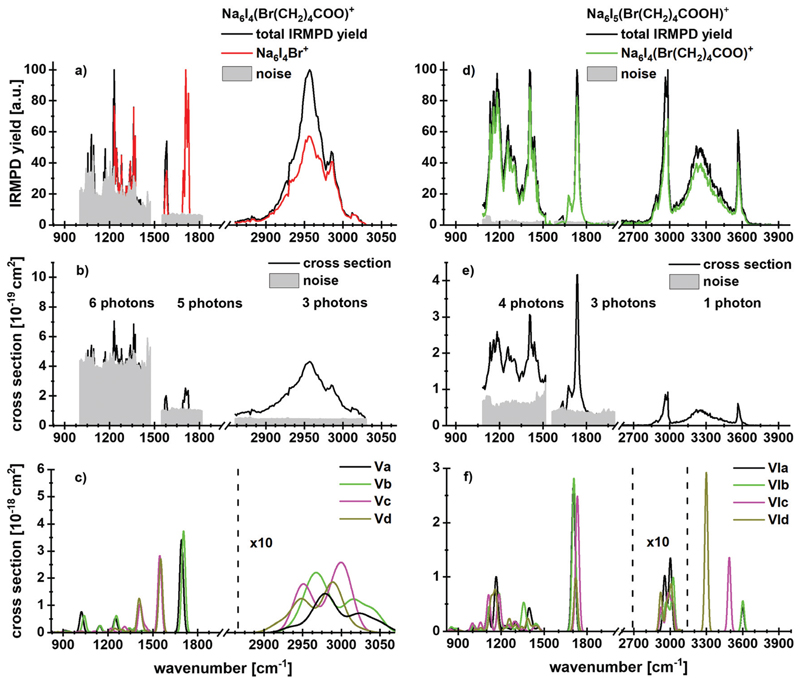
Experimental IRMPD yield spectra of (a) Na_6_I_4_(Br(CH_2_)4COO)^+^ and (d) Na_6_I_5_(Br(CH_2_)_4_COOH)^+^ with contribution of the most intense fragmentation products. (b and e) Multiphoton photodissociation cross sections derived from (a and d). (c and f) Calculated spectra in harmonic approximation for Na_6_I_4_(Br(CH_2_)_4_COO)^+^ and Na_6_I_5_(Br(CH_2_)_4_COOH)^+^, respectively, for 833–3846 cm^−1^. Here, the regions measured in detail are shown. The overview spectra are provided in Fig. S3 (ESI†). The theoretical spectra are scaled with a factor of 0.97.

Fig. 4 shows experimental and calculated IR spectra of Na_6_I_4_(Br(CH_2_)_4_COO) and Na_6_I_5_(Br(CH_2_)_4_COOH). The spectra for Na_6_I_4_(Br(CH_2_)_4_COO) were measured with 20 s (region IR1), 10 s (region IR2) and 3 s (region IR3) irradiation time, the irradiation times for Na_6_I_5_(Br(CH_2_)_4_COOH)^+^ were 10 s (region IR1), 10 s (region IR2) and 3 s (region IR3). For 5-bromovalerate complexed with salt, several different fragmentation patterns are observed (Table 1), where the dominant fragment Na_6_I_4_Br is consistent with valerolactone elimination. In lower yields, Na_2_I^+^ and Na_4_I_2_(Br(CH_2_)_4_COO)^+^ are observed. Na_4_I_2_Br^+^ appears with less intensity and may be created during secondary reactions. The most stable structure in Fig. 2d, isomer Va, shows that lactone formation is again energetically preferred.

In contrast to 4-bromobutyrate, both the valerolactone and 5-bromovalerate seem to be present in the experimental ion population, evidenced by the peaks in the C–O stretch region. The lactone structure (isomers **Va,b**) is identified by the peak at 1712 cm^−1^. The presence of 5-bromovalerate (isomers **Vc,d**) is responsible for the absorption at 1575 cm^−1^. Since the Na_6_I_4_Br^+^ fragment appears also following this excitation, we conclude that lactone is formed from 5-bromovalerate activated by IR light. The kinetics (Fig. S12, ESI†) shows that lactone elimination is again a primary process. The kinetic fit requires absorption of multiple photons. The spectral features in the C–H stretch region around 3000 cm^−1^ and the fingerprint region of 850–1480 cm^−1^ cannot be assigned to specific isomers. The theoretical and experimental cross sections are comparable in the C–H and fingerprint region, while a difference of more than an order of magnitude is observed for the C–O modes.

The spectrum and fragmentation behavior changes for 5-bromovaleric acid, see Fig. 4d-f. As also evident from the kinetics, Fig. S13 (ESI†), the most intense fragmentation channel is the evaporation of HI from the cluster, with a low reaction energy of 72 kJ mol^−1^, or 27 kJ moP^1^ if lactone is formed in parallel. Thus, at least one photon is needed for decomposition at 2540–3840 cm^−1^. The Na_6_I_5_^+^ fragment appears as the second most abundant fragment, with a calculated reaction energy of 105 kJ mol^−1^. Interestingly, also Na_6_I_4_Br^+^ is observed, which according to the fit is a secondary product. It is thus seamlessly explained as HI elimination followed by lactone formation and loss.

The absorption centered at 3564 cm^−1^ originates from a free O–H vibration of the acid, the broad absorption at 3250 cm^−1^ again from the OH…I^−^ interaction. The doublet around 2970 cm^−1^ and a weak transition at 2886 cm^−1^ can be assigned to C–H vibrations, based on the comparison with the spectrum in Fig. 4a and the calculations.

For the C–O absorption observed at 1720 cm^−1^, Na_6_I_4_(Br(CH_2_)_4_-COO)^+^ with HI elimination is the most intense fragmentation channel while Na_6_I_5_^+^ appears with lower intensity. In the 1100–1500 cm^−1^ region, HI elimination is again the favored dissociation pathway. The absorption centered at 1400 cm^−1^ results from a CH_2_ bending mode and indicates the presence of isomer **VIa** or **VIb**, showing a local absorption in this region. Two further peaks with similar intensities, induced by coupled C–C and C–O vibrations, again point out that more than one isomer is needed to interpret the IR spectrum.

The measurements were repeated with bromoalkanoates and bromoalkanoic acids with longer aliphatic chains. These experiments did not yield clear evidence for lactone formation. For 8-bromooctanoate, loss of [NaI]_2_ is the preferred fragmentation channel (Fig. S14, ESI†), while no fragment that would correspond to lactone formation is present in the investigated IR region. Upon doping the salt cluster with intact 8-bromo-octanoic acid, the dominant fragment channels are loss of HI competing with loss of the complete molecule.

In the case of 11-bromoundecanoate (Fig. S15 and S16, ESI†), loss of [NaI]_2_ again is the dominant reaction channel. Interestingly, the C-Br bond becomes activated in these smaller salt clusters, evidenced by Na_4_I_2_(I(CH_2_)_10_COO)^+^ and Na_2_(I(CH_2_)_10_COO)^+^, the dominant secondary fragments. Obviously, I^−^ replaces Br^−^ in an S_N_2 reaction.^66^ This is the reverse of the standard S_N_2 reaction, most likely mediated by the interaction with the salt. A fragment that could point to lactone formation, Na_2_Br^+^, appears late with low intensity. However, it is more likely formed by loss of Na_2_I(I(CH_2_)_10_COO) from Na_4_I_2_(Br(CH_2_)_10_COO)^+^, which has already rearranged to Na_4_IBr(I(CH_2_)_10_COO)^+^.

### Reaction paths for lactone formation

**Fig. 5:**
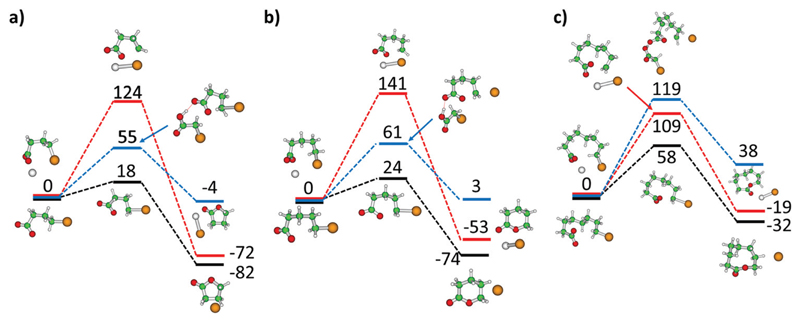
Reaction path to form lactones from bromoalkanoate Br(CH_2_)_m_COO^−^ in the gas phase (black curve), in the presence of a sodium ion NaBr(CH_2_)_m_COO (red curve) and in the Br(CH_2_)COOH·Br(CH_2_)_m_COO^−^ model complex (blue curve) for (a) *m* = 3, (b) *m* = 4, (c) *m* = 7. Calculated at the B3LYP/def2TZVP level, energies are given in kJ mol^−1^. Color code: Na grey, Br brown, C green, O red, H white.

The experimental observation of Br^−^ ions, either bare, complexed to bromoalkanoic acid, or embedded in a [NaI]_x_ cluster, is evidence for lactone formation. To get a better idea how and when lactone formation is feasible in the experiment, we calculated the reaction path for *m* = 3, 4 and 7 in three model environments as shown in Fig. 5: bare bromoalkanoate, bromoalkanoate complexed with Na^+^, and a simplified bromoalkanoate-bromoalkanoic acid complex. For bare bromoalkanoate, the barrier increases monotonically along the *m* = 3, 4, 7 series from 18 kJ mol^−1^ to 58 kJ mol^−1^. To get closer to the relevant barrier of lactone formation in the experiment, we repeated this calculation with a simplified model of the studied complex, the complex Br(CH_2_)COOH·Br(CH_2_)_m_COO^−^ in which the aliphatic chain of the neutral molecule was shortened to reduce both the conformational flexibility and the number of electrons. Here, one can expect a barrier of about 61 and 119 kJ mol^−1^ for lactone formation with *m* = 4 and 7, respectively. Due to the high barrier for the longer chain, lactone formation in the isolated complex can be ruled out, since direct dissociation is both energetically preferred, Table 1, and with a loose transition state also mechanistically favored. For *m* = 7, fragments involving lactone elimination therefore most likely result from isomers **IId,e** formed in the ESI process. The kinetics for *m* = 7, 10 in Fig. S6 and S7 (ESI†) also reveal that the potential lactone elimination takes place much less efficiently for longer alkano-ate chains than for the complex with *m* = 4, Fig. S8 (ESI†), further hinting that the ring has to be present before irradiation. Lactone elimination is also induced by ambient black-body infrared radiation for *m* = 4 with a very small rate of 0.006 s^−1^, see Fig. S9 (ESI†) for the BIRD kinetics.

The lactone formation path for bromobutyrate and the bromobutyrate-sodium complex NaBr(CH_2_)_3_COO is provided in Fig. 5a. Compared to butyrolactone formation in the gas phase with a small barrier of 18 kJ mol^−1^ and exothermicity of 82 kJ mol^−1^, the presence of the Na^+^ counterion increases the barrier to 124 kJ mol^−1^, since the interaction between the CO_2_^−^group, Br and Na stabilizes the reactant compared to the transition state structure. The reaction energy stays almost unaffected by the presence of Na^+^. This suggests that lactone formation takes place during the electrospray process, since complexation of bromobutyrate with the salt cluster increases the barrier and slows down the reaction.

For 5-bromovalerate, lactone formation faces a higher barrier and is less exothermic than for 4-bromobutyrate (Fig. 5a and b). Compared to the barrier in the gas phase of 24 kJ mol^−1^, the barrier increases to 141 kJ mol^−1^ when the ion complexes with Na^+^. This can explain the presence of both isomers in the experimental mixture. Lactones may be formed in the ESI source, with a slightly higher reaction barrier compared to 4-bromovalerate. The reaction is then hindered as soon as the organic ion is complexed with salt.

We found transition states for lactone formation for 8-bromooctanoate and 11-bromoundecanoate in the gas phase, with barriers of 58 and 44 kJ mol^−1^, respectively, about twice the value of 5-bromovalerate. We consider it highly unlikely to happen in the salt environment during IRMPD, as the long bromoalkyl chain has to be in the correct orientation to form the lactone ring, which is exceedingly improbable.

## Conclusion

IRMPD of negatively charged complexes with *n*-bromoalkanoic acids (*n* = 5, 8) clustered with the corresponding n-bromo-alkanoates, as well as positively charged sodium iodide clusters doped with the intact acids and alkanoates (*n* = 4, 5) was investigated experimentally and theoretically. The anionic complexes showed that lactone formation can be initiated by IR light for *n* = 5, and some fragments indicating formation of rings were also observed for *n* = 8. However, the formation via IR light is not expected for the latter case due to steric effects and a concomitant higher barrier, implying that a small fraction of clusters already contained lactone before irradiation. The failure to generate these complexes for *n* = 4 by ESI suggests that lactone formation is even more facile for this small system, making the complex unstable under the experimental conditions.

The fragmentation behavior of the cationic salt clusters including an attached intact bromoalkanoic acid molecule shows the loss of HI, [NaI]_x_ and the intact acid. The situation changes for bromoalkanoates embedded in salt environment. Here, lactone evaporation was observed with 4-bromobutyrate, and the spectra suggest that butyrolactone is already present before irradiation of the cluster with laser light, formed during the ESI process. 5-Bromovalerate is slightly more stable, the valerolactone is formed both before and during laser irradiation. Experiment and calculations show that the salt environment tends to stabilize bromoalkanoates against lactone formation by increasing the barrier with respect to the free bromoalkanoate in the gas phase for 4-bromobutyrate and 5-bromovalerate.

In a real sea-salt aerosol, similar reaction mechanisms may be operative, albeit not driven by multiple infrared photons. Due to their larger size and the high-pressure environment in the atmosphere, they are efficiently thermalized. Reactions in the aerosol will be either thermally activated, or photochemi-cally by visible or ultraviolet light. Moreover, sea-salt aerosols consist mainly of NaCl, which will behave differently from the NaI clusters studied here. Nevertheless, our work shows that the barriers for thermally activated lactone formation from bromoalkanoates via an intramolecular S_N_2 reaction depend sensitively on the local environment of the carboxylate group and the bromine atom involved in the reaction. We suggest that this applies also to S_N_2 reactions in real sea-salt aerosols.

## Supplementary Material

Supplementary Information

## Figures and Tables

**Table 1:** Intensity of fragmentation channels of *n*-bromoalkanoic acid and *n*-bromoalkanoates, *n* = 5, 7, of the anionic complexes Br(CH_2_)_m_COOH·Br(CH_2_)_m_COO^−^ and in salt environment for *m* = 3, 4 along with reaction energies Δ*E* calculated at the B3LYP/def2TZVP level of theory with respect to the most stable parent ion (Fig. 2). For the alkanoate/alkanoic acid-salt clusters, only the fragmentation channels with a branching ratio >4% are shown, see Table S1 (ESI) for the complete listing

Parent ion	Products	Δ*E* [kJ mol^−1^]	IR1^*a*^ [%]	IR2^*b*^ [%]	IR3^*c*^ [%]
Br(CH_2_)_4_COOH·Br(CH_2_)_4_COO^−^	Br^−^ + (CH_2_)_4_COO·Br(CH_2_)_4_COOH	114	–	1.1	5.2
	Br(CH_2_)_4_COOH·Br^−^ + (CH_2_)_4_OCO	42	–	98.5	94.8
	Br(CH_2_)_4_COO^−^ + Br(CH_2_)_4_COOH	90	–	0.4	–
Br(CH_2_)_7_COOH·Br(CH_2_)_7_COO^−^	Br^−^ + (CH_2_)_7_COO·Br(CH_2_)_7_COOH	61	–	11.6	28.6
	Br(CH_2_)_7_COOH·Br^−^ + (CH_2_)_7_OCO	34	–	7.5	–
	Br(CH_2_)_7_COO^−^ + Br(CH_2_)_7_COOH	76	–	80.9	71.4
Na_6_I_4_(Br(CH_2_)3COO)^+^	Na_6_I_4_Br^+^ + (CH_2_)_3_OCO	99	88.2	100	94.5
	Na_2_Br^+^ + (NaI)_4_(CH_2_)_3_COO	174	4.4	–	–
	Na_2_I^+^ + Na_4_I_3_(Br(CH_2_)_3_COO)	178	4.9	–	3.0
Na_6_I_5_(Br(CH_2_)_3_COOH)^+^	Na_6_I_4_(Br(CH_2_)_3_COO)^+^ + HI	14/77^d^	38.5	29.5	26.5
	Na_6_I_5_^+^ + Br(CH_2_)_3_COOH	99	60.3	70.5	73.5
Na_6_I_4_(Br(CH_2_)_4_COO)^+^	Na_6_I_4_Br^+^ + (CH_2_)_4_OCO	104	71.0	90.7	69.0
	Na_2_I^+^ + Na_4_I_3_(Br(CH_2_)_4_COO)	222	29.1	–	–
	Na_4_I_2_(Br(CH_2_)_4_COO)^+^ + (NaI)_2_	183	–	9.3	28.6
Na_6_I_5_(Br(CH_2_)4COOH)^+^	Na_6_I_4_(Br(CH_2_)_4_COO)^+^ + HI	27/72^d^	85.0	89.9	75.0
	Na_6_I_4_Br^+^ + HI + (CH_2_)_4_OCO	130	6.5	–	1.4
	Na_6_I_5_^+^ + Br(CH_2_)_4_COOH	105	8.5	8.3	23.0

a 833–1500 cm^−1^ for salt environment.

b 1000–2200 cm^−1^ for anionic complexes; 1600–1800 cm ^−1^ for salt environment.

c 2200–4000 cm^−1^ for anionic complexes; 2600-4000 cm^−1^ for salt environment.

d Reaction energies for a cluster with/without lactone formation.
