# Supplementary material for: Evidence for lactone formation during infrared multiple photon dissociation spectroscopy of bromoalkanoate doped salt clusters
Source: Phys Chem Chem Phys. Author manuscript; Available in PMC 2020 Nov 8. (PMC7116335; doi:10.1039/d0cp00272k)
Supplement: Supplementary Information [file EMS102274-supplement-Supplementary_Information.pdf]

Supporting Information for the Manuscript:

Evidence for Lactone Formation during Infrared Multiple Photon Dissociation Spectroscopy of Bromoalkanoate Doped Salt Clusters

Nina K. Bersenkowitsch, Milan Ončák, Jakob Heller, Tobias F. Pascher, Christian van der Linde, and Martin K. Beyer

Institut für Ionenphysik und Angewandte Physik, Universität Innsbruck, Technikerstraße 25, 6020 Innsbruck, Austria

E-mail: milan.oncak@uibk.ac.at; martin.beyer@uibk.ac.at

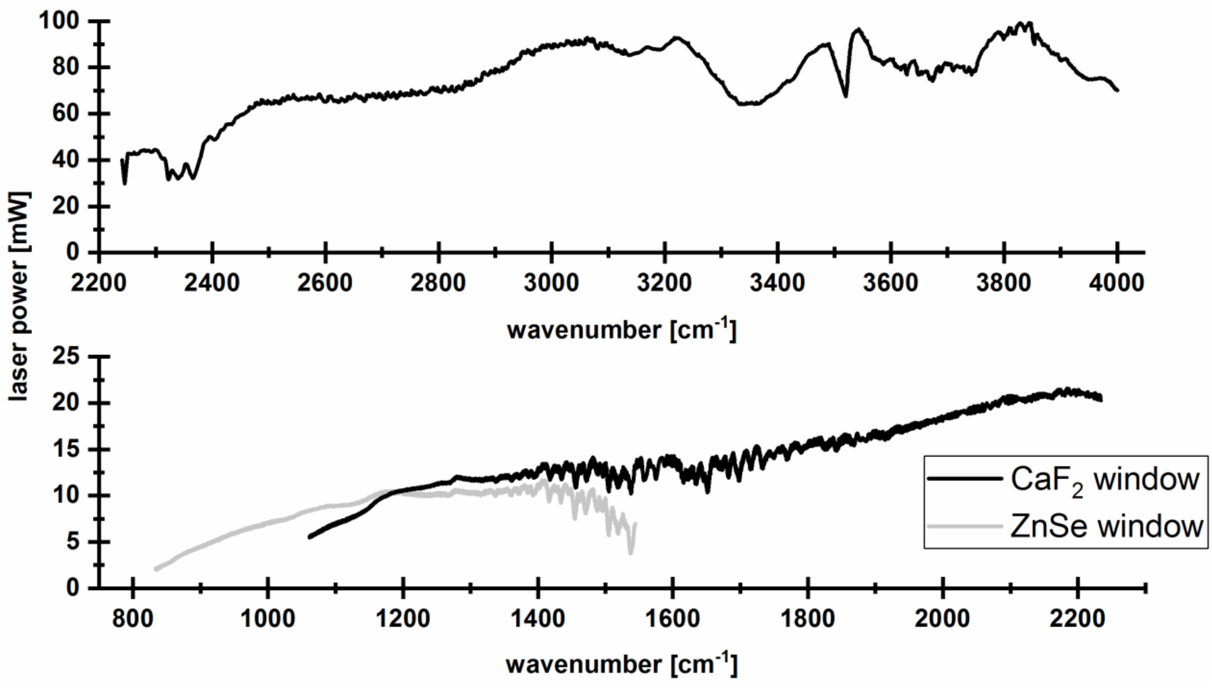

Figure S1: Laser energies for both tunable laser systems used. The spectra were measured at the distance from the laser to the ICR cell (3.65m). Due to the transmittance of the window in front of the ICR cell, two windows with different materials (CaF_2, ZnSe) were used.

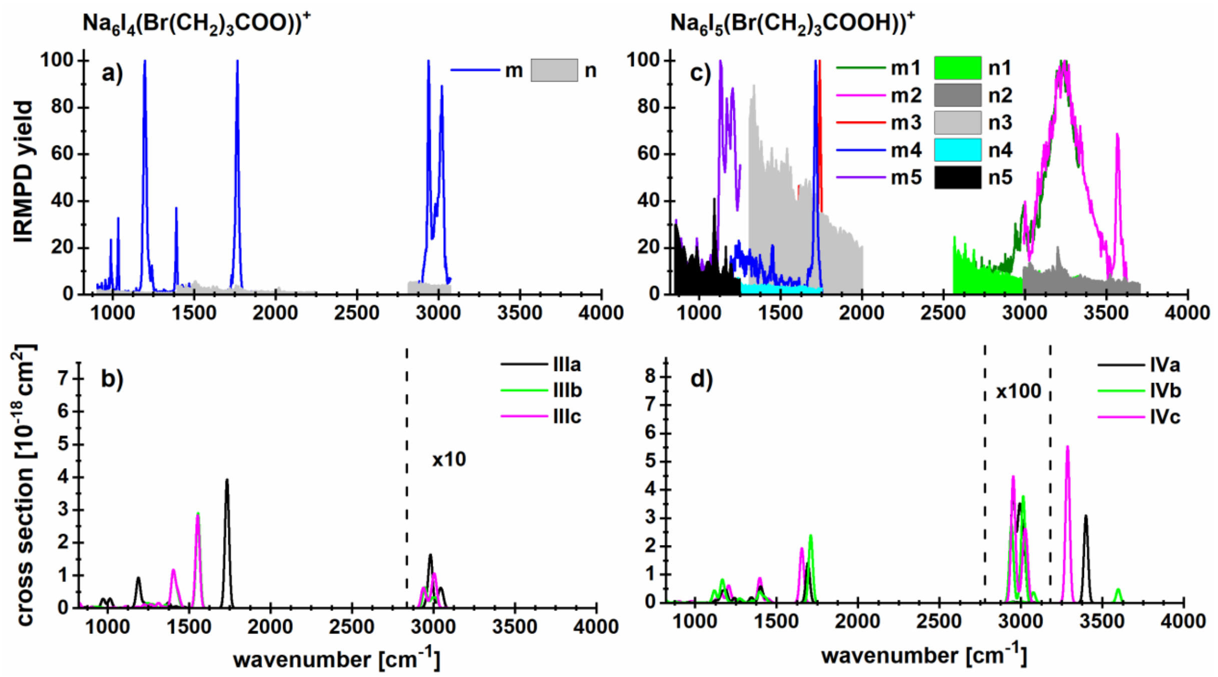

Figure S2: **a,c)** Overview spectra of $Na_6I_4(Br(CH_2)_3COO)^+$ and $Na_6I_5(Br(CH_2)_3COOH)^+$. The total IRMPD yield of measurements **mX** with corresponding noise **nX** are displayed here, which are all normalized separately to their maximum. The corresponding theory is shown in **b,d)**.

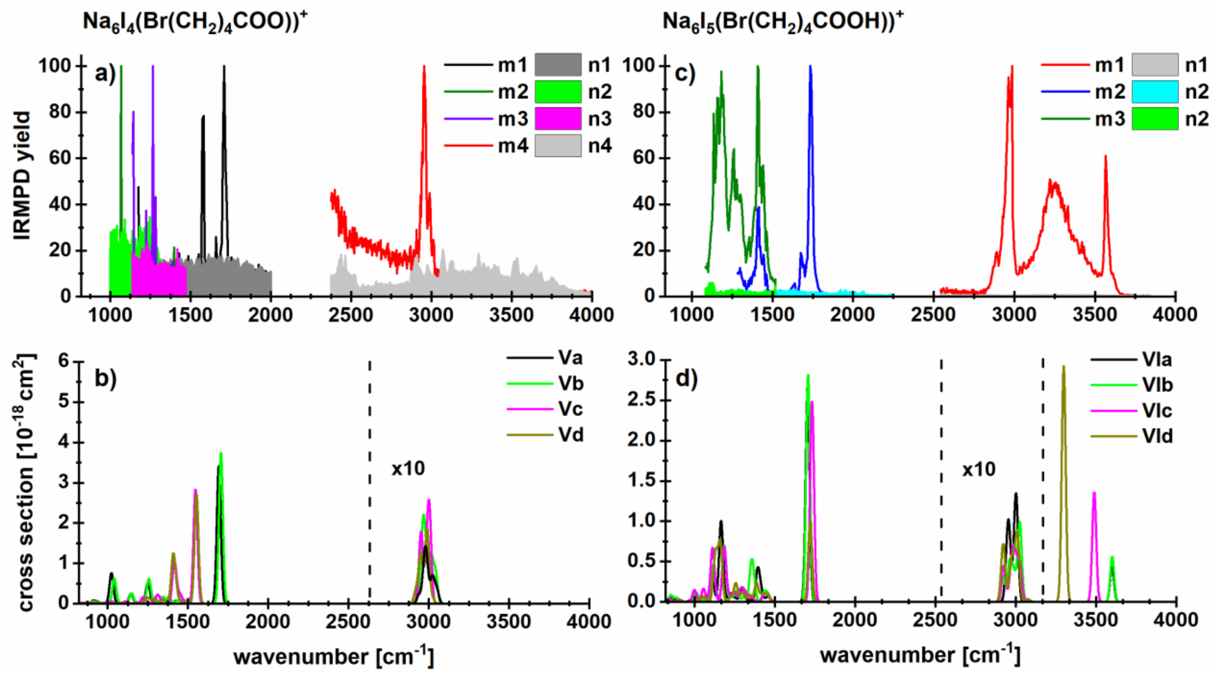

Figure S3: **a,c)** Overview spectra of $Na_6I_4(Br(CH_2)_4COO)^+$ and $Na_6I_5(Br(CH_2)_4COOH)^+$. The total IRMPD yield of measurements **mX** with corresponding noise **nX** are displayed here, which are all normalized separately to their maximum. The corresponding theory is shown in **b,d)**.

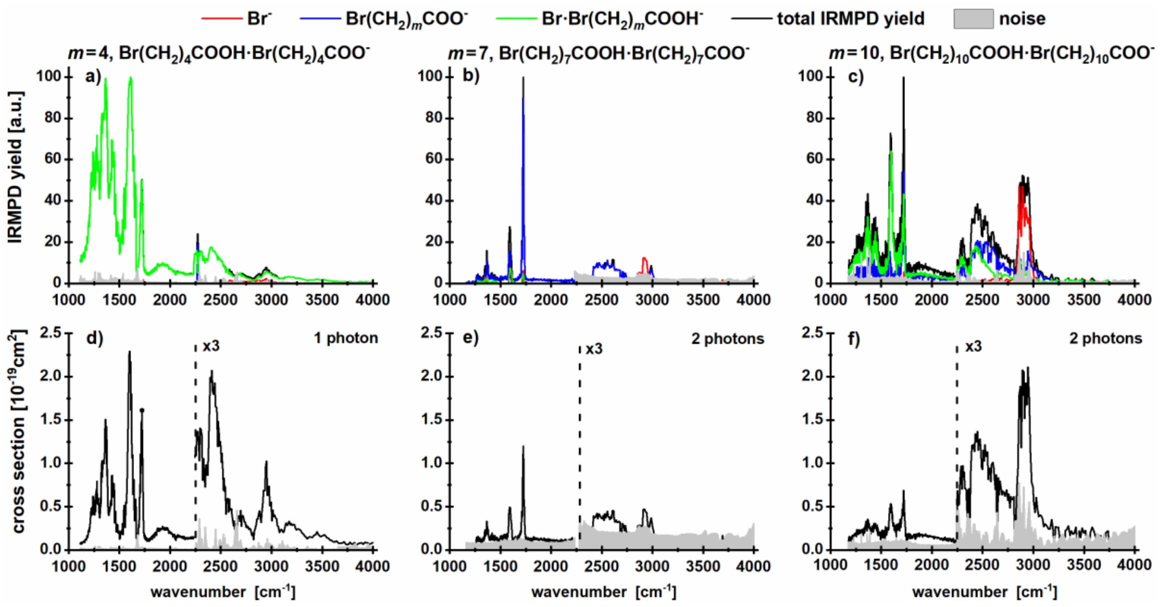

Figure S4: (a-c) IRMPD yield spectra and **(d-f)** experimental cross sections of the cluster $Br(CH_2)_mCOO.Br(CH_2)_mCOOH^-$ with m = 4 (column 1), m = 7 (column 2) and m = 10 (column 3).

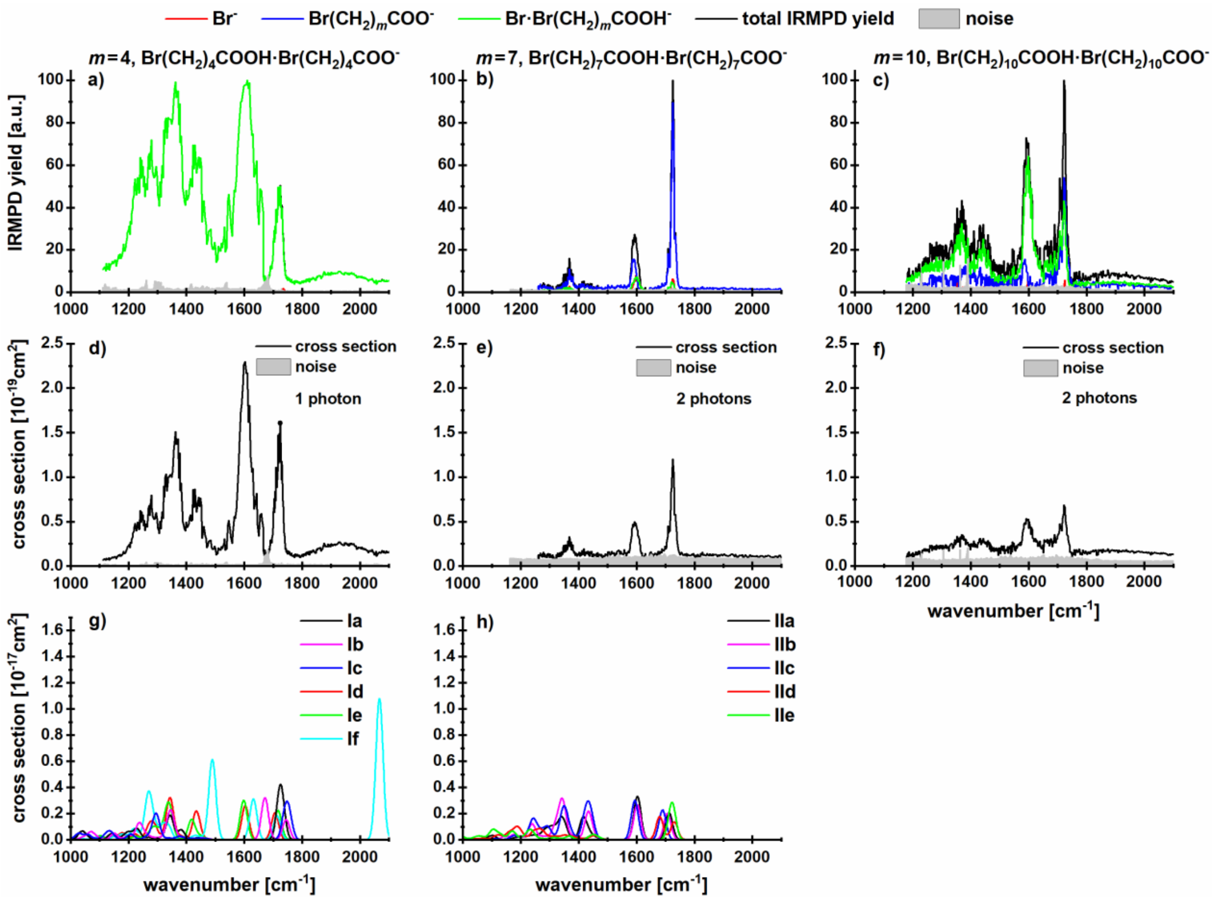

Figure S5: (a-c) IRMPD yield spectra, **(d-f)** experimental and **(g-h)** theoretical cross sections of the cluster $Br(CH_2)_mCOO.Br(CH_2)_mCOOH^-$ with m = 4 (column 1), m = 7 (column 2) and m = 10 (column 3) in the low-energy part of the total spectrum.

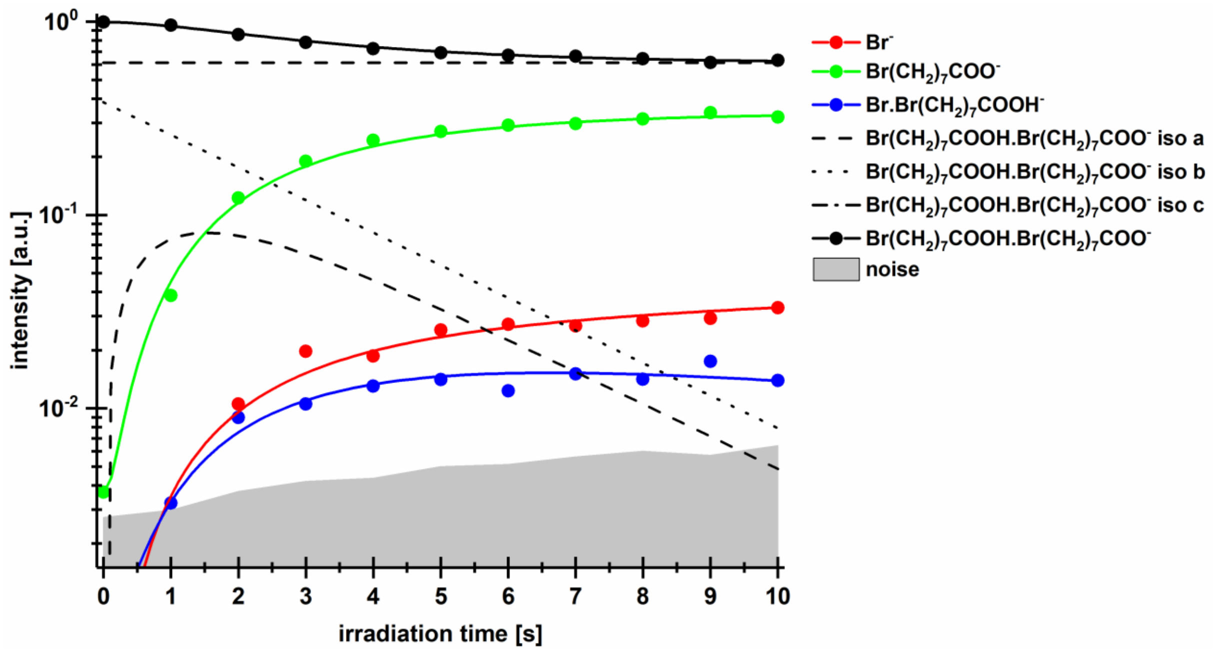

Figure S6: Kinetics of the cluster $Br(CH_2)_7COOH.Br(CH_2)_7COO^-$ at 1723 cm^{-1}.

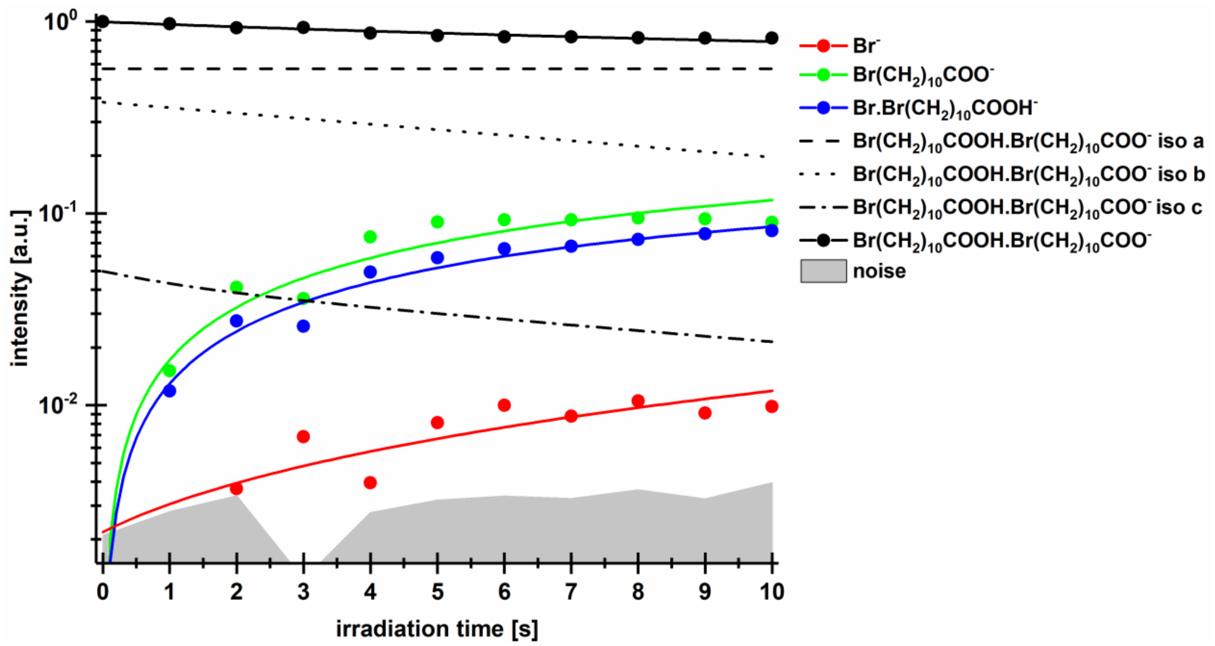

Figure S7: Kinetics of the cluster $Br(CH_2)_{10}COOH.Br(CH_2)_{10}COO^-$ at 1724 cm^{-1}.

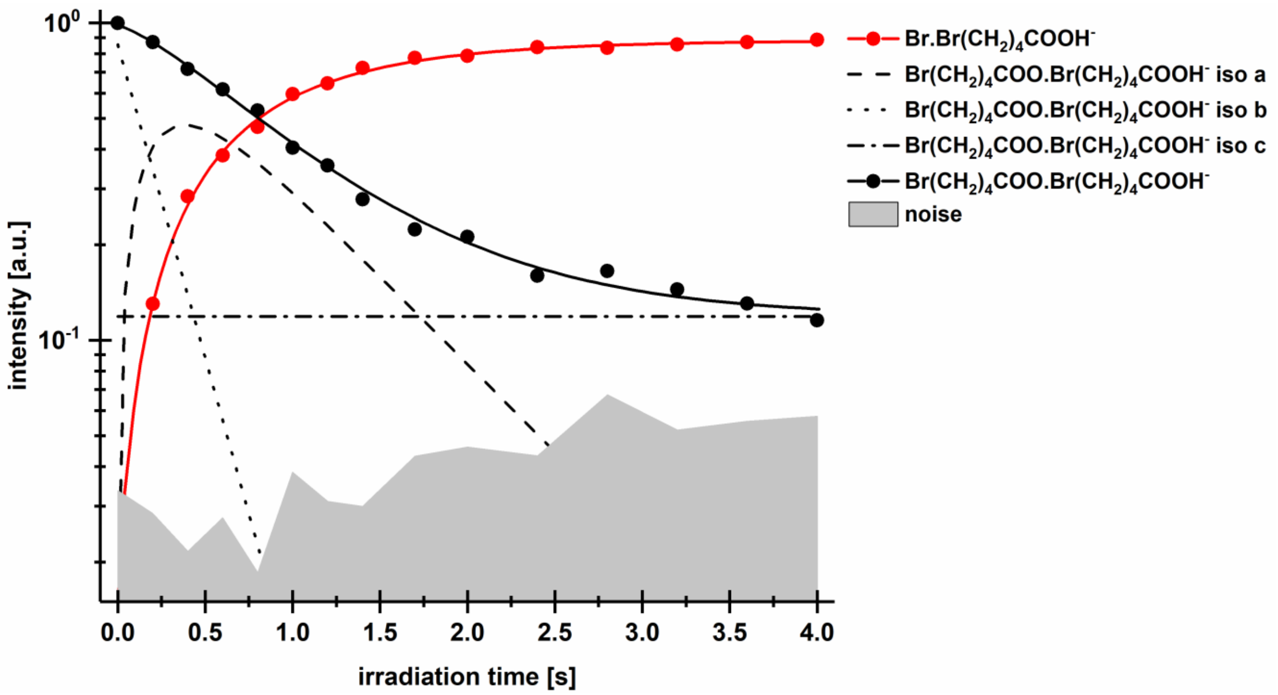

Figure S8: Kinetics of the cluster $Br(CH_2)_4COOH.Br(CH_2)_4COO^-$ at 1725 cm^{-1}.

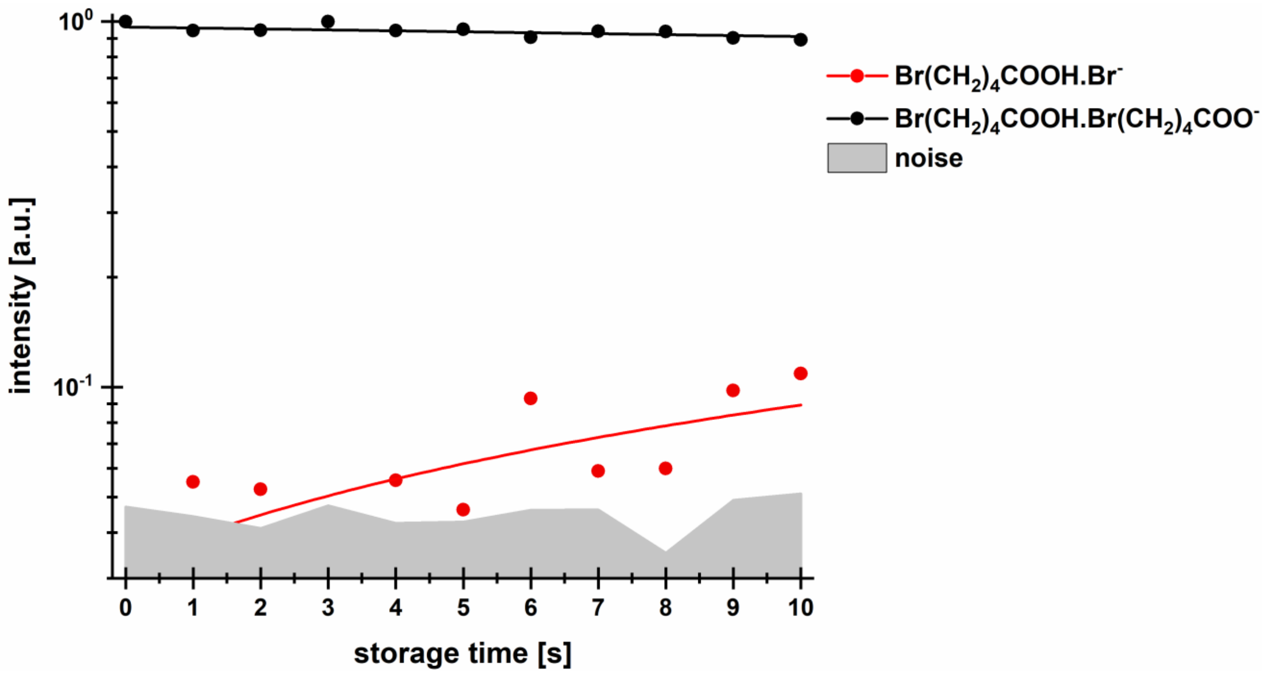

Figure S9: BIRD kinetics of the cluster $Br(CH_2)_4COOH.Br(CH_2)_4COO^-$.

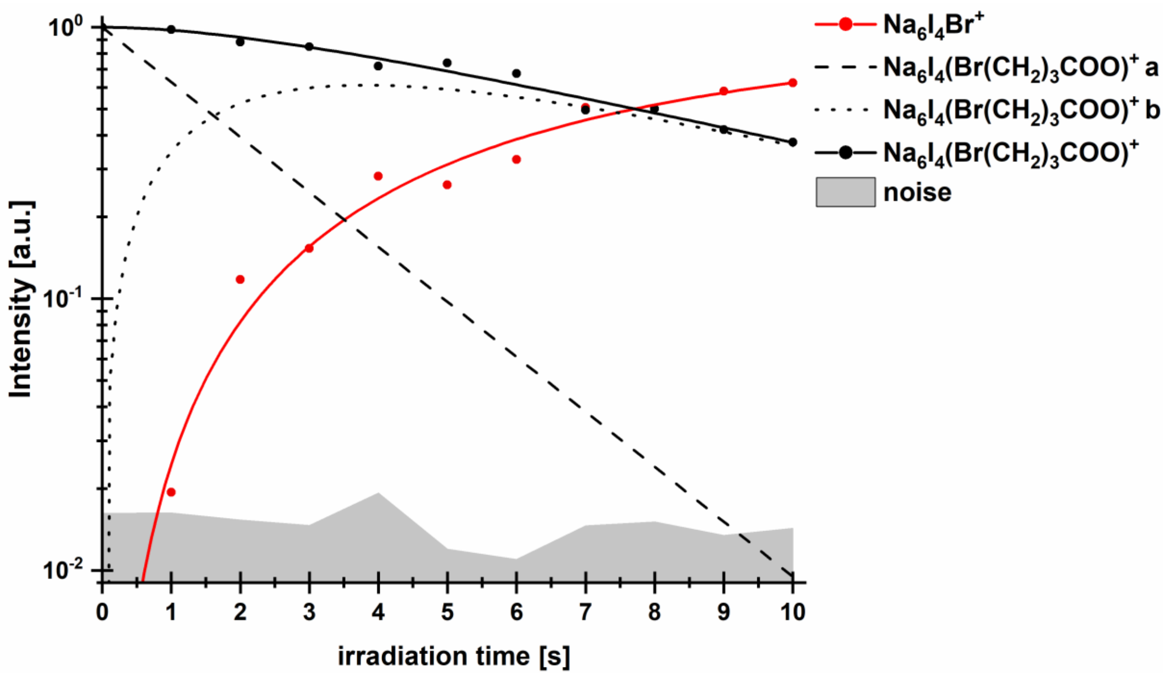

Figure S10: Kinetics of the cluster $Na_6I_4(Br(CH_2)_3COO)^+$ at 2940 cm^{-1}.

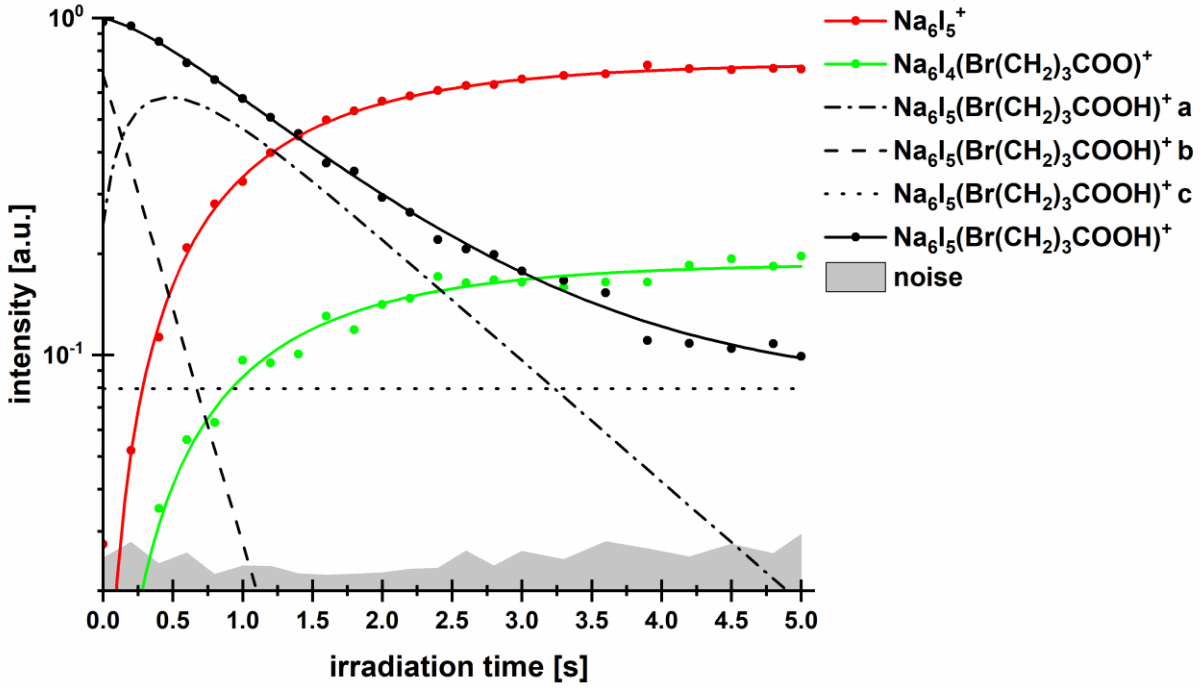

Figure S11: Kinetics of the cluster $Na_6I_5(Br(CH_2)_3COOH)^+$ at 3226 cm^{-1}.

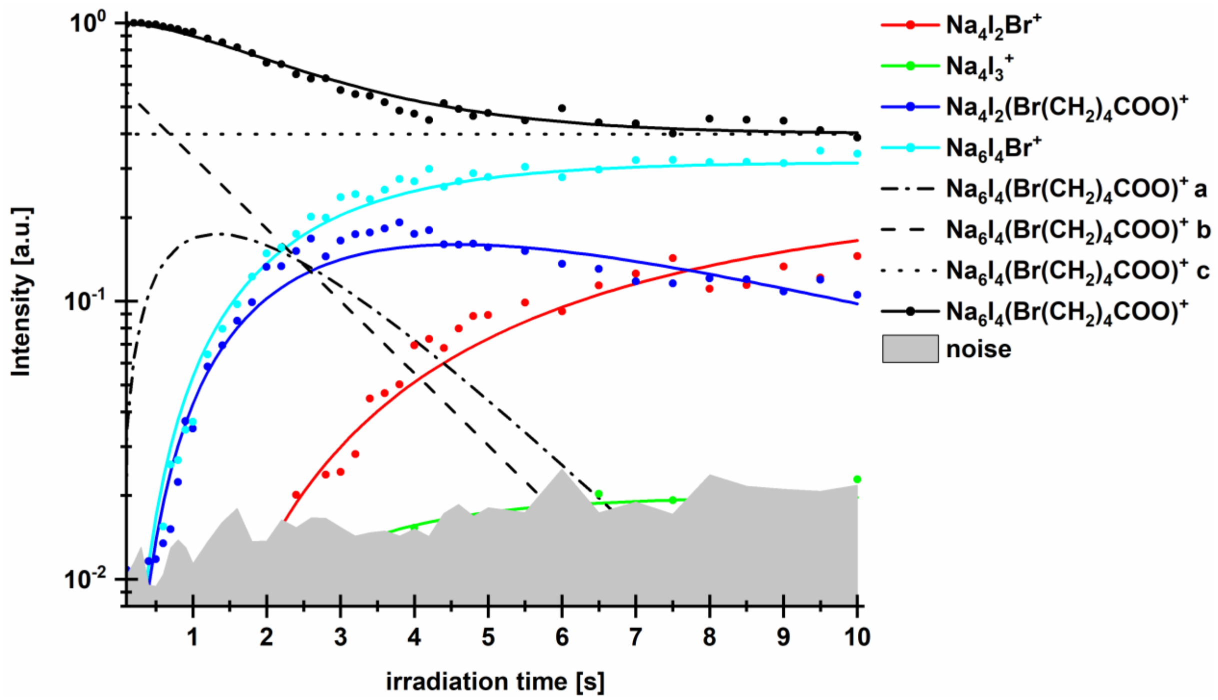

Figure S12: Kinetics of the cluster $Na_6I_4(Br(CH_2)_4COO)^+$ at 2940 cm^{-1}.

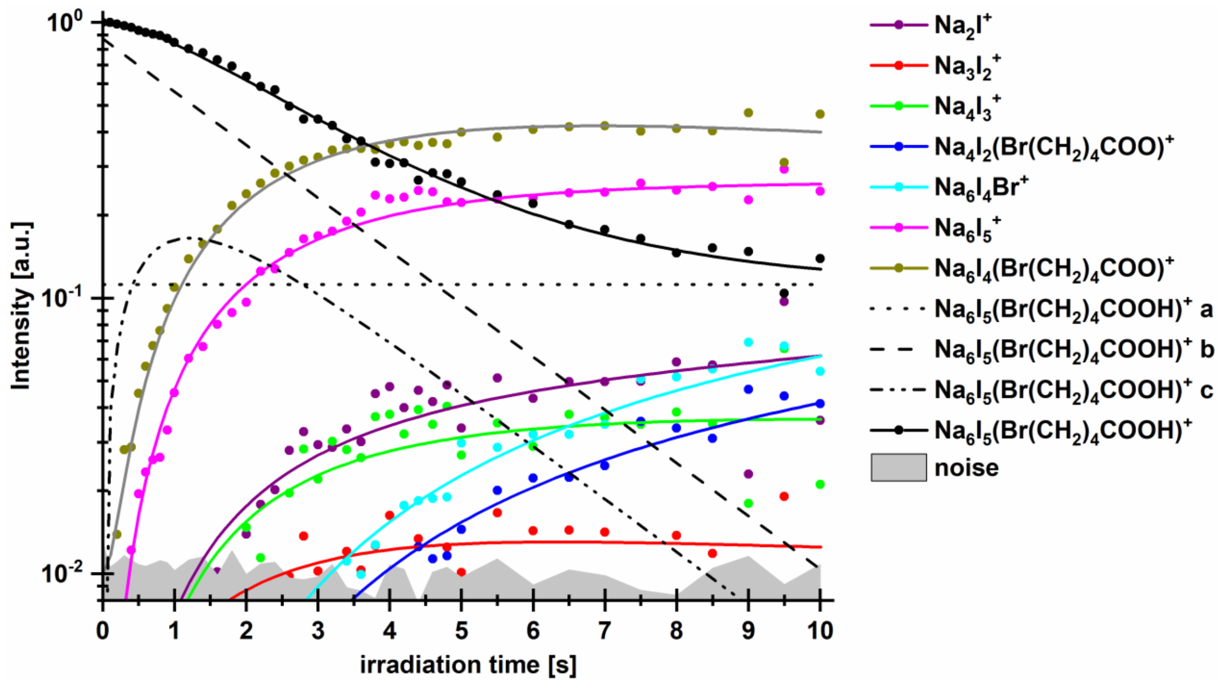

Figure S13: Kinetics of the cluster $Na_6I_5(Br(CH_2)_4COOH)^+$ at 2985 cm^{-1}.

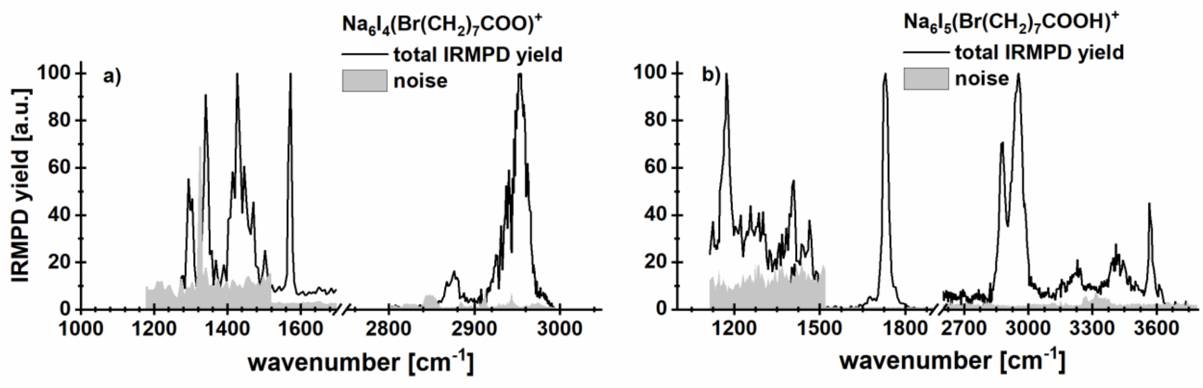

Figure S14: IRMPD spectrum for **a)** $Na_6I_4(Br(CH_2)_7COO)^+$ (irradiation times 15s, 10s, 3s) and **b)** $Na_6I_5(Br(CH_2)_7COOH)^+$ (irradiation times 10s, 5s, 3s), respectively, from 833-3846 cm^{-1}.

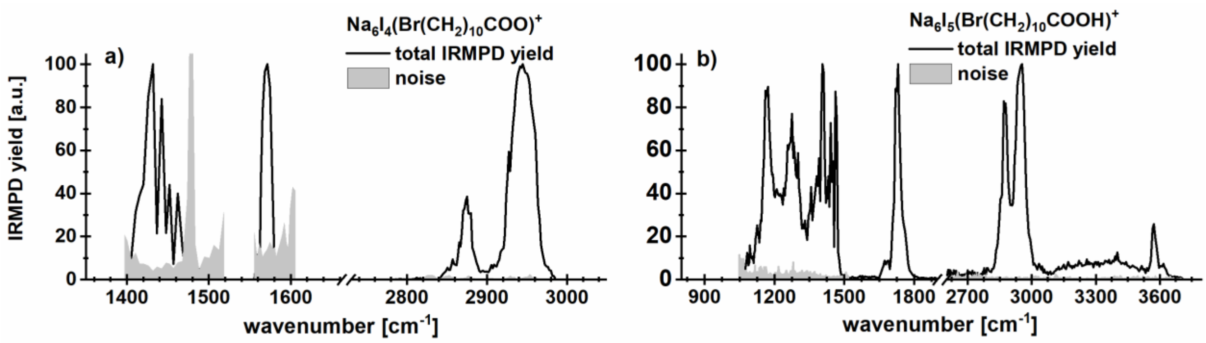

Figure S15: IRMPD spectrum for **a)** $Na_6I_4(Br(CH_2)_{10}COO)^+$ (irradiation times 3s, 20s, 15s) and **b)** $Na_6I_5(Br(CH_2)_{10}COOH$ (irradiation times 3s, 5s, 15s) respectively, from 833-3846 cm^{-1}.

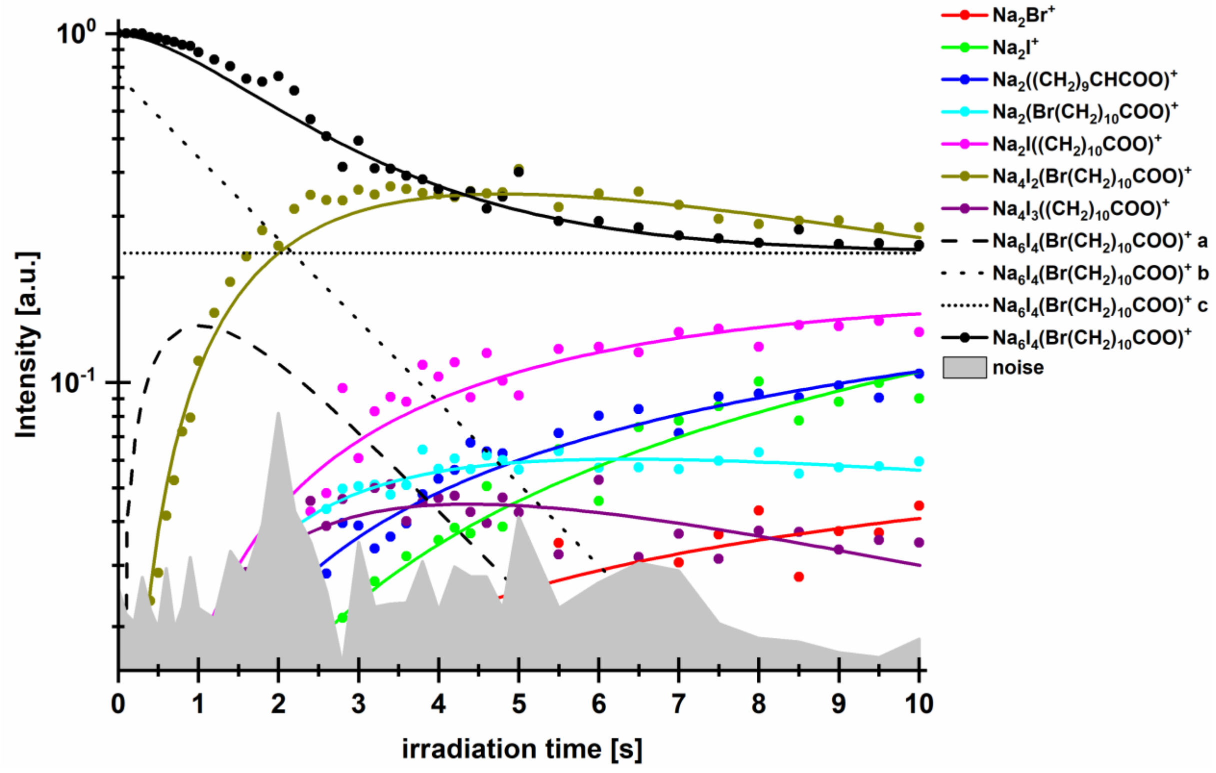

Figure S16: Kinetics of the cluster $Na_6I_4(Br(CH_2)_{10}COO)^+$ at 2945 cm^{-1}.

Table S1: Intensity of various channels of *n*-bromoalkanoic acid and *n*-bromoalkanoates, *n* = 4,5, in salt environment and of the anionic complexes $Br(CH_2)_mCOOH.Br(CH_2)_mCOO^-$ for *m* = 4,7,10 along with the reaction energies Δ*E*. Calculated at the B3LYP/def2TZVP level of theory.

parent ion	product	Δ*E* [kJ/mol]	IR1 [%]	IR2 [%]	IR3 [%]
IR2 1000–2200 cm^{-1} IR3 2200–4000 cm^{-1}					
$Br(CH_2)_4COOH.Br(CH_2)_4COO^-$	$Br^- + (CH_2)_4COO.Br(CH_2)_4COOH$	114	-	1.1	5.2
	$Br(CH_2)_4COOH.Br^- + (CH_2)_4OCO$	42	-	98.5	94.8
	$Br(CH_2)_4COO^- + Br(CH_2)_4COOH$	90	-	0.4	-
$Br(CH_2)_7COOH.Br(CH_2)_7COO^-$	$Br^- + (CH_2)_7COO.Br(CH_2)_7COOH$	61	-	11.6	28.6
	$Br(CH_2)_7COOH.Br^- + (CH_2)_7OCO$	34	-	7.5	-
	$Br(CH_2)_7COO^- + Br(CH_2)_7COOH$	76	-	80.9	71.4
IR1 833–1500 cm^{-1} IR2 1600–1800 cm^{-1} IR3 2600–4000 cm^{-1}					
$Na_6I_4(Br(CH_2)_3COO)^+$	$Na_6I_4Br^+ + (CH_2)_3OCO$	99	88.2	100	94.5
	$Na_4I_2Br^+ + (NaI)_2(CH_2)_3COO$	160	0.4	-	0.6
	$Na_2Br^+ + (NaI)_4(CH_2)_3COO$	174	4.4	-	-
	$Na_4I_3^+ + Na_2I(Br(CH_2)_3COO)$	167	2.0	-	1.2
	$Na_3I_2^+ + Na_3I_2(Br(CH_2)_3COO)$	203	-	-	0.6
	$Na_2I^+ + Na_4I_3(Br(CH_2)_3COO)$	178	4.9	-	3.0
$Na_6I_5(Br(CH_2)_3COOH)^+$	$Na_6I_4(Br(CH_2)_3COO)^+ + HI$	14	38.5	29.5	26.5
	$Na_6I_5^+ + (Br(CH_2)_3COOH$	99	60.3	70.5	73.5
	$Na_2I^+ + HI + Na_4I_3(Br(CH_2)_3COO)$	191	0.9	-	-
	$Na_4I_3^+ + HI + Na_2I(Br(CH_2)_3COO)$	181	0.3	-	-
$Na_6I_4(Br(CH_2)_4COO)^+$	$Na_6I_4Br^+ + (CH_2)_4OCO$	104	71.0	90.7	69.0
	$Na_4I_2Br^+ + (NaI)_2((CH_2)_4COO)$	194	-	-	2.2
	$Na_4I_3^+ + Na_2I(Br(CH_2)_4COO)$	206	-	-	0.3
	$Na_2I^+ + Na_4I_3(Br(CH_2)_4COO)$	222	29.1	-	-
	$Na_4I_2(Br(CH_2)_4COO)^+ + (NaI)_2$	183	-	9.3	28.6
$Na_6I_5(Br(CH_2)_4COOH)^+$	$Na_6I_4(Br(CH_2)_4COO)^+ + HI$	27	85.0	89.9	75.0
	$Na_4I_2(Br(CH_2)_4COO)^+ + (NaI)_2 + HI$	210	0.1	1.9	0.3
	$Na_6I_4Br^+ + HI + (CH_2)_4COO$	130	6.5	-	1.4
	$Na_6I_5^+ + Br(CH_2)_4COOH$	105	8.5	8.3	23.0
	$Na_4I_3^+ + HI + Na_2I(Br(CH_2)_4COO)$	232	-	-	0.1

Cartesian coordinates of optimized ions and molecules (in Å, calculated at the B3LYP/def2TZVP level unless noted otherwise) along with electronic energies (in Hartree) including zero point energy

Ia
E = -5840.752116
C -4.554766 0.364560 0.872815
O -5.729218 -0.466280 0.628585
C -5.934230 -1.102154 -0.535867
C -5.065188 -0.754015 -1.735716
C -3.779667 0.018564 -1.441074
C -4.053377 1.066150 -0.369193
O -6.848286 -1.886750 -0.622770
O -0.587506 2.261012 0.503405
C 0.621563 2.672279 0.129737
C 1.747842 1.663855 0.305228
C 2.972718 1.971683 -0.549775
C 4.188959 1.086478 -0.237842
C 3.934761 -0.380197 -0.527100
Br 5.578450 -1.470078 -0.267272
O 0.797759 3.793426 -0.293117
Br -0.807829 -0.849473 1.124828
H 5.039945 1.424425 -0.834394
H 1.351086 0.662219 0.128865
H 3.253619 3.016730 -0.407877
H 2.711370 1.873701 -1.608472
H 4.472977 1.203018 0.811837
H 2.014298 1.680123 1.369027
H -0.613674 1.285522 0.751341
H -4.879089 -1.688595 -2.266137
H -5.721500 -0.163767 -2.385676
H -3.403314 0.477979 -2.358015
H -2.997292 -0.650749 -1.074374
H -3.142772 1.609712 -0.112854
H -4.792919 1.796804 -0.718188
H -4.879034 1.064533 1.641993
H -3.770302 -0.274733 1.283348
H 3.198888 -0.831409 0.130922
H 3.652287 -0.555709 -1.561907

Ib
E = -5840.744891
O -3.441417 -0.407932 1.881286
C -3.070864 0.428619 0.903402
C -3.085248 -0.030291 -0.523676
C -3.974578 -1.237371 -0.810812
C -3.705715 -2.284346 0.262831
C -4.030385 -1.709654 1.624385
O -2.682356 1.533776 1.242507
O -1.772943 3.455258 -0.448121
C -0.450301 3.667836 -0.477488
O 0.014760 4.527337 -1.187388
C 0.417478 2.781781 0.402780
C 1.210677 1.754998 -0.422312
C 2.240968 1.014543 0.439927
C 2.964405 -0.042713 -0.367431
Br 4.464436 -0.849746 0.672237
H 1.739332 0.534911 1.283978
H -0.180578 2.270242 1.158458
H 1.718502 2.278334 -1.237060
H 0.532696 1.025721 -0.872986
H 2.957896 1.731499 0.851433
H 1.115993 3.447714 0.916210
H -2.035930 2.733389 0.173933
H -3.311574 0.832795 -1.149810
H -2.045212 -0.333361 -0.746205
H -3.724860 -1.631249 -1.796479
H -5.034824 -0.956668 -0.820142
H -4.316193 -3.180548 0.118378
H -2.654003 -2.579721 0.203381
H -3.661307 -2.340744 2.432754
H -5.110618 -1.584004 1.752925
H 2.313177 -0.873034 -0.635976
H 3.447257 0.365225 -1.251827
Br -0.172800 -2.026494 -1.200147

Ic
E = -5840.740040
O 2.040375 3.075200 -0.187747
C 0.729726 3.038279 -0.520416
O 0.250222 3.953270 -1.141292
C -0.024062 1.814379 -0.049111
C -1.487921 1.794705 -0.475709
C -2.258250 0.644050 0.186580
C -3.664976 0.575867 -0.366848
Br -4.821861 -0.713766 0.614259
H -3.684918 0.229042 -1.396234
H -4.196720 1.521029 -0.285452
H -1.737595 -0.298712 0.002428
H -2.293689 0.800819 1.268300
H -1.531253 1.690331 -1.562889
H -1.957525 2.752952 -0.236129
H 0.472422 0.912611 -0.430613
H 0.068058 1.768574 1.042119
H 2.316894 2.271896 0.292892
O 3.338100 0.726129 0.802121
C 2.781637 -0.112457 1.727622
C 2.854780 -1.592262 1.474934
C 4.055948 -2.048825 0.647022
C 4.149652 -1.168557 -0.593927

C 4.307871 0.279778 -0.185946
H 4.173452 0.955989 -1.029371
H 5.294866 0.465131 0.251519
H 5.007797 -1.439917 -1.216373
H 3.238988 -1.295025 -1.187606
H 3.919038 -3.093882 0.363517
H 4.980473 -1.988778 1.234821
H 1.940743 -1.817350 0.900717
H 2.780254 -2.093493 2.439652
O 2.195188 0.391583 2.649692
Br 0.534753 -1.739389 -1.428507

Id
E = -5840.738474
C 4.356718 0.244418 -0.231040
C 2.914795 0.517335 0.219022
C 2.281568 1.700275 -0.505746
C 0.837467 2.026021 -0.061253
O 0.299304 2.972124 -0.717529
C 4.938578 -0.934778 0.516133
O 0.334578 1.367422 0.861993
C -2.785611 1.879294 -0.175645
C -4.011201 1.061862 0.216588
C -3.847634 -0.426867 -0.117968
C -5.066997 -1.207264 0.320820
C -2.951535 3.374293 0.074918
O -4.020898 3.864262 0.391220
O -1.869300 4.103958 -0.087985
H 4.976386 1.128620 -0.058398
H 2.878949 2.606098 -0.358057
H 2.298990 -0.371896 0.058606
H 2.885775 0.703770 1.295521
H 4.383764 0.045691 -1.305934
H 2.264590 1.529934 -1.586792
H -1.004106 3.561191 -0.312106
H -2.563844 1.752310 -1.241462
H -1.887836 1.536955 0.348588
H -4.191965 1.181848 1.288906
H -4.896618 1.464521 -0.282274
H -3.700033 -0.552273 -1.193985
H -2.955770 -0.824686 0.371504
Br 6.816026 -1.339603 -0.022202
H 4.997172 -0.762649 1.587349
H 4.398938 -1.858256 0.324864
Br -4.948772 -3.144674 -0.128648
H -5.208307 -1.188721 1.398089
H -5.976332 -0.872420 -0.171247

Ie
E = -5840.738158
C 3.889164 0.005690 -0.546645
C 3.515366 1.352902 0.086665
C 2.957488 2.348701 -0.940180
C 2.536563 3.651044 -0.271475
O 3.226577 4.650647 -0.321167
C 4.103260 -1.027545 0.537361
O 1.395301 3.623667 0.393604
Br -6.746318 -0.782215 -0.300666
C -5.114109 0.318873 0.016466
C -3.862515 -0.518475 0.157574
C -2.635686 0.370605 0.403530
C -1.342807 -0.427828 0.552211
H 4.784467 0.102661 -1.167411
H 3.706657 2.590200 -1.696099
H 2.754242 1.172493 0.847724
H 4.393018 1.788107 0.576068
H 3.076934 -0.328270 -1.196181
H 2.090106 1.897819 -1.428390
H 0.876109 2.736640 0.316643
H -5.072431 0.991433 -0.835797
H -5.342249 0.884564 0.915719
H -3.986940 -1.226571 0.981356
H -3.715233 -1.111665 -0.749073
H -2.511313 1.083741 -0.413772
H -2.794172 0.970845 1.305758
Br 4.723312 -2.777817 -0.197664
H 4.883713 -0.746640 1.239826
H 3.179081 -1.248096 1.063404
C -0.066923 0.431513 0.707409
H -1.394812 -1.109783 1.403859
H -1.194220 -1.053252 -0.336497
O 0.879593 -0.071526 1.334373
O -0.102290 1.568963 0.142805

If
E = -5840.735749
C 4.463311 0.458276 -0.310893
C 3.353899 1.297125 0.335777
C 2.609894 2.156952 -0.695369
C 1.542762 3.037200 -0.047535
O 1.715210 4.233556 0.097464
C 5.113538 -0.445317 0.713635
O 0.449311 2.427170 0.356806
Br -7.395980 -0.260086 0.019340
C -5.622274 -1.164962 0.125634
C -4.463266 -0.207999 -0.043955
C -3.126225 -0.957267 0.039086
C -1.918814 -0.040518 -0.126650
H 5.216794 1.111671 -0.759372
H 3.306654 2.818863 -1.211966
H 2.634533 0.633820 0.818564
H 3.786395 1.946203 1.104951
H 4.039733 -0.149338 -1.113636
H 2.138832 1.493040 -1.423693
H 0.427681 1.383860 0.159392
H -5.625408 -1.648650 1.098689

H -5.657784 -1.919343 -0.655602
H -4.546631 0.300946 -1.007997
H -4.511055 0.566563 0.725997
H -3.052337 -1.477138 0.999074
H -3.081456 -1.738779 -0.722858
Br 6.648859 -1.468696 -0.037136
H 5.541731 0.105908 1.546800
H 4.432212 -1.205343 1.084779
C -0.561940 -0.783302 -0.137126
H -1.991792 0.513883 -1.069222
H -1.895744 0.719473 0.659460
O 0.471083 -0.029594 -0.070148
O -0.555409 -2.012308 -0.224886

IIa
E = -6076.532745
C -4.527008 -0.676485 1.361134
C -3.008132 -0.781818 1.227910
C -2.255422 -0.393853 2.500917
C -0.736951 -0.469956 2.352411
C -0.105415 0.551603 1.375733
O -0.785557 1.605601 1.166190
C -5.274023 -1.038996 0.075989
O 1.011461 0.273412 0.912293
O -0.529336 3.450288 -0.568609
C 0.588729 4.149710 -0.639388
O 0.687579 5.128352 -1.355721
C 1.764166 3.641175 0.183037
C 2.620382 2.659559 -0.629872
C 3.764774 2.067989 0.189789
C 4.591310 1.044902 -0.588252
C 5.694838 0.398821 0.251505
H -2.577664 -1.046256 3.323394
H -2.526379 0.629017 2.773387
H -0.264217 -0.294517 3.325443
H -0.420684 -1.468420 2.037955
H -2.740841 -1.807921 0.944673
H -2.669620 -0.132247 0.416720
H -4.873106 -1.326085 2.174747
H -4.791067 0.346019 1.652632
H 5.038067 1.519900 -1.470349
H 3.920776 0.263764 -0.959792
H 4.421257 2.870678 0.551028
H 3.331637 1.578594 1.066161
H 3.009795 3.170006 -1.517528
H 1.982620 1.838564 -0.962405
H 2.362516 4.507154 0.475184
H 1.404603 3.132531 1.078677
H -0.515080 2.679265 0.119321
C 6.531848 -0.620178 -0.532500
H 6.354070 1.179096 0.650245
H 5.238117 -0.091886 1.117451
C 7.590542 -1.248726 0.347437
H 5.881248 -1.399599 -0.936514
H 7.008252 -0.130987 -1.386136
Br 8.727622 -2.551082 -0.635621
H 8.292945 -0.518734 0.741243
H 7.164487 -1.822039 1.166348
C -6.798773 -0.949816 0.218559
H -4.941473 -0.375565 -0.729290
H -4.996801 -2.054527 -0.229530
C -7.492762 -1.301968 -1.079678
H -7.137110 -1.625415 1.008625
H -7.083992 0.060355 0.522869
Br -9.473298 -1.237826 -0.924106
H -7.257796 -0.605711 -1.880164
H -7.277688 -2.316249 -1.405544

IIb
E = -6076.532509
C -4.763792 0.405308 -0.030974
C -6.221551 0.190519 0.381134
C -4.191069 1.747268 0.427393
C -2.736582 1.969394 0.010127
C -2.171441 3.306373 0.475595
C -0.729184 3.607069 0.007958
O -0.212263 2.869301 -0.845433
O -0.209709 4.626015 0.563776
O 1.933341 5.765410 -0.150446
C 3.040099 5.057319 -0.242369
O 4.097757 5.556288 -0.583141
C 2.912343 3.583293 0.120724
C 4.157581 2.759903 -0.185021
C 4.001037 1.293037 0.217312
C 5.222626 0.435094 -0.112978
C 5.061122 -1.032622 0.287979
H -4.811594 2.557879 0.025556
H -2.799001 4.129106 0.113251
H -2.114407 1.158678 0.402747
H -2.644399 1.905926 -1.077057
H -4.274942 1.818552 1.519012
H -2.190252 3.381012 1.567250
H 1.084780 5.215983 0.110514
H 2.672733 3.535090 1.189598
H 2.029631 3.178986 -0.385169
H 4.375328 2.823935 -1.255594
H 5.024332 3.196782 0.319154
H 3.798892 1.230966 1.293679
H 3.118028 0.874827 -0.278017
H -4.681835 0.330764 -1.121105
H -4.150745 -0.407546 0.374512
H 5.426050 0.493894 -1.188238
H 6.106009 0.850591 0.385697
C -6.788564 -1.158340 -0.079523
H -6.303135 0.266109 1.471497
H -6.834853 1.001704 -0.027526

C -8.231070 -1.323248 0.349450
H -6.719273 -1.237129 -1.167379
H -6.188697 -1.972957 0.334067
Br -9.001508 -3.060100 -0.227176
H -8.347344 -1.316625 1.430062
H -8.884179 -0.573787 -0.089895
C 6.289988 -1.888601 -0.044104
H 4.855676 -1.092524 1.362819
H 4.180346 -1.447612 -0.214357
C 6.081285 -3.330811 0.365770
H 6.496838 -1.839665 -1.116187
H 7.169808 -1.487315 0.465409
Br 7.657141 -4.471184 -0.038027
H 5.930097 -3.441984 1.436260
H 5.258981 -3.800557 -0.167326

IIc
E = -6076.526698
C 5.863698 -0.369281 -0.396250
C 6.145700 -0.747358 1.044731
C 4.869816 -0.788430 1.901554
C 4.210586 0.572737 2.132986
C 2.950394 0.509330 3.005217
C 1.724237 -0.190424 2.396528
C 1.100070 0.509108 1.186254
C 1.659128 0.110286 -0.175646
O 1.156413 0.748574 -1.204733
Br 7.522884 -0.462828 -1.499421
O 2.513133 -0.753524 -0.310165
H 1.180486 1.597317 1.275863
H 0.021976 0.327368 1.138216
H 1.971025 -1.218543 2.123797
H 0.964487 -0.252512 3.181689
H 2.661183 1.532262 3.272438
H 3.205508 0.012053 3.949689
H 3.959787 1.029702 1.173716
H 4.935662 1.240928 2.614523
H 4.149270 -1.457179 1.429032
H 5.131964 -1.224449 2.872859
H 6.867268 -0.044400 1.472929
H 6.618731 -1.732512 1.062648
H 5.522972 0.652987 -0.517130
H 5.156867 -1.036114 -0.879010
H 0.397122 1.440145 -0.968965
O -0.592488 2.503780 -0.804035
C -1.712399 2.282364 -0.242943
O -2.069495 1.238797 0.327560
C -2.681649 3.486048 -0.289314
C -4.081520 3.215550 0.255721
H -2.204372 4.293028 0.275660
H -2.724224 3.837913 -1.324985
H -4.626330 4.162766 0.361696
C -4.896347 2.255703 -0.610612
H -3.986954 2.786492 1.257131
C -6.265978 1.915552 -0.019242
H -5.039211 2.692153 -1.607480
H -4.303156 1.351099 -0.747157
C -7.050762 0.836478 -0.784617
H -6.142326 1.597840 1.022706
H -6.866326 2.831238 0.019988
H -8.123474 1.030634 -0.685531
H -6.830092 0.914221 -1.855245
C -6.831038 -0.614300 -0.318299
H -7.525456 -1.262756 -0.859152
C -5.422715 -1.141227 -0.520210
H -7.092462 -0.694086 0.740714
Br -5.324319 -3.068438 -0.038024
H -5.103775 -1.102585 -1.558131
H -4.673757 -0.664545 0.103909

IId
E = -6076.522133
C 2.986175 -0.920145 -0.458234
Br 3.137367 -2.745802 -1.263036
C 4.357622 -0.346429 -0.170393
C 4.306323 1.067696 0.436305
C 3.763129 2.166910 -0.500416
C 2.303280 2.550900 -0.245424
C 1.778141 3.593324 -1.233133
C 0.362799 4.074510 -0.917806
C -0.714456 3.033609 -1.148088
O -0.604359 2.076128 -1.877927
O -1.830659 3.318819 -0.465745
C -4.251650 0.584640 0.213679
O -3.492636 -0.072268 1.073303
C -2.033363 -0.188622 0.911555
C -1.723637 -1.672834 0.835866
C -2.011205 -2.357454 -0.513652
C -3.317954 -1.980856 -1.230695
C -4.640468 -2.299786 -0.495716
C -5.737250 -1.241032 -0.716600
C -5.648608 0.007232 0.199713
H -6.343947 0.771372 -0.147240
H -1.729740 0.359577 0.021378
H -1.563825 0.256143 1.786718
H -2.270148 -2.162482 1.646857
H -0.665585 -1.779809 1.087324
H -1.190339 -2.124740 -1.198156
H -1.973728 -3.441947 -0.362755
H -3.278067 -0.918481 -1.480319
H -3.330703 -2.487245 -2.201366
H -4.462865 -2.409895 0.575649
H -5.017069 -3.266886 -0.841175
H -5.717087 -0.914886 -1.760828
H -6.722793 -1.685223 -0.551123
H -5.913478 -0.278634 1.219025

H -2.502111 2.606933 -0.595561
H 0.097758 4.930019 -1.550056
H 0.284209 4.423411 0.113912
H 2.443837 4.464214 -1.232180
H 1.796977 3.183931 -2.247102
H 2.212624 2.930515 0.777057
H 1.669107 1.663617 -0.273830
H 3.883848 1.854997 -1.545742
H 4.376840 3.068712 -0.391786
H 5.330533 1.319334 0.729266
H 3.712893 1.035884 1.354387
H 4.870051 -1.008869 0.531541
H 4.954146 -0.331012 -1.088652
H 2.387047 -1.040978 0.443660
H 2.431741 -0.359327 -1.204299
Br 1.207672 -0.097818 2.775606
O -3.887315 1.482129 -0.524169

IIe
E = -6076.521664
C -4.379615 -0.299693 0.165498
Br -6.152728 -1.133764 0.571940
C -4.525195 0.800472 -0.867117
C -3.173054 1.424348 -1.249804
C -2.507120 2.245575 -0.144568
C -1.135213 2.772838 -0.564219
C -0.489123 3.666726 0.494160
C 0.916789 4.191097 0.127852
C 1.986101 3.134039 0.273641
O 2.665517 2.990981 1.266203
O 2.094420 2.354248 -0.809159
O 3.959394 0.220095 -0.379895
C 4.189396 -0.770247 -1.299161
O 5.281266 -0.963684 -1.771970
C 2.974327 -1.614280 -1.567222
C 3.113980 -2.972999 -0.823807
C 2.643127 -3.015378 0.642591
C 3.065110 -1.861184 1.559484
C 4.581150 -1.601116 1.720126
C 4.928144 -0.103914 1.831130
C 5.061649 0.603156 0.481815
H 4.997000 1.683491 0.598334
H 2.041231 -1.125466 -1.280694
H 2.953480 -1.810478 -2.640133
H 4.151443 -3.307553 -0.916934
H 2.509584 -3.697507 -1.373549
H 1.550572 -3.024858 0.638847
H 2.975309 -3.972013 1.065437
H 2.571444 -0.958863 1.203079
H 2.633740 -2.045870 2.547943
H 5.142412 -2.037278 0.888437
H 4.944721 -2.116447 2.613759
H 4.166393 0.401870 2.429533
H 5.881842 0.033730 2.352313
H 5.989464 0.341547 -0.024574
H 2.700140 1.606735 -0.615652
H 1.190441 5.006761 0.797441
H 0.912314 4.564593 -0.898885
H -1.129287 4.537735 0.672112
H -0.429084 3.131455 1.446672
H -1.228552 3.340598 -1.499052
H -0.494621 1.915182 -0.779027
H -2.371618 1.620373 0.741999
H -3.161369 3.079311 0.146468
H -3.333529 2.066949 -2.123782
H -2.484939 0.629139 -1.549319
H -4.987523 0.378897 -1.763271
H -5.208107 1.570501 -0.492771
H -3.738252 -1.112200 -0.164405
H -4.033144 0.054305 1.130181
Br -0.710671 -1.356647 -0.408349

IIIa
E = -5046.251263
I 0.615968 -1.696006 -0.743963
Na -0.478319 0.704950 1.086627
I 1.913405 2.615652 0.742880
Na 3.132658 0.055142 -0.375084
Na -0.115541 3.929346 -0.913535
I -2.387664 2.047422 -1.295607
Na -1.744360 -0.752014 -2.486100
Br -3.804112 -2.202355 -1.375151
Na -1.996785 -2.862847 0.660918
Na -4.303507 -0.003949 0.278553
I -2.611455 -0.695087 2.671416
O 5.235434 -0.440425 -0.791188
C 6.308572 -0.852697 -0.401352
O 6.474412 -1.292994 0.843625
C 7.862121 -1.679861 1.081140
C 8.466338 -1.867348 -0.312756
C 7.602754 -0.941769 -1.174145
H 7.420182 -1.289397 -2.188734
H 8.014288 0.069528 -1.244420
H 8.356434 -2.902271 -0.637545
H 9.525047 -1.618028 -0.335195
H 7.841222 -2.579927 1.689329
H 8.330174 -0.871956 1.644389

IIIb
E = -5046.227069
Na -2.232950 -1.161918 1.425072
I -4.278940 0.890675 0.586455
Na -3.123999 3.500802 -0.095794
I 0.472657 -1.335461 2.773222
Na 0.831701 -0.623207 -2.586848
I 3.269651 -1.444299 -1.027823

Na 1.044277 -3.024500 0.311572
Na 2.772397 0.134883 1.487314
I -1.447678 -2.360598 -1.405296
Na -2.493502 0.572026 -1.916052
Br 3.119496 3.089126 1.098880
C 3.208088 3.445019 -0.871814
C 2.096101 2.757180 -1.634810
C 0.696128 3.280897 -1.329422
C -0.461479 2.390098 -1.764008
O -1.629805 2.791587 -1.495067
O -0.243052 1.285140 -2.328017
H 4.191220 3.086021 -1.157237
H 3.176362 4.529606 -0.928668
H 2.153880 1.684557 -1.447432
H 2.322078 2.891381 -2.698361
H 0.538458 4.265315 -1.777849
H 0.576741 3.424973 -0.251686

IIIc
E = -5046.225349
Na -1.053143 -0.747521 -0.750373
I -0.112053 -0.668046 2.412272
Na 1.526527 1.445608 0.789990
I 4.057527 -0.072720 0.354170
Na 2.329687 -0.419654 -2.072462
Na 2.180143 -2.380722 0.991933
I 0.820134 -2.964349 -1.645609
Na -3.638861 1.494398 -1.516205
I -4.193639 -1.002929 -0.109141
Na -2.993257 -1.341727 2.548960
Br -0.102568 3.980921 0.816386
C 0.218772 4.976484 -0.884462
C -0.448553 4.359208 -2.096250
C 0.151748 3.039370 -2.598361
C -0.268026 1.785123 -1.840518
O -1.491510 1.517370 -1.736898
O 0.636471 1.025571 -1.373719
H 1.300432 5.024457 -0.974667
H -0.172547 5.964903 -0.665449
H 1.240390 3.102704 -2.612770
H -0.176424 2.889107 -3.630800
H -0.340990 5.103764 -2.892548
H -1.520757 4.250325 -1.924724

IVa
E = -5344.656409
Na 1.462610 -2.009875 1.770239
I 2.438371 -2.131877 -1.263181
Na 0.908650 0.288894 -2.546910
Na 4.003528 0.295336 -0.148793
I 3.055530 0.358785 2.715595
Na 1.598966 2.697384 1.549266
I 2.473563 2.642122 -1.433377
I -1.932521 -0.715597 -2.512400
Na -0.480467 -3.139804 -1.387110
I -1.368791 -3.026599 1.511499
Na -3.198909 -1.039249 0.208765
Br -5.328654 0.979196 0.719862
C -4.756689 2.532447 1.841886
C -3.278903 2.836527 1.731795
C -2.834686 3.318843 0.354678
C -1.339612 3.411359 0.207133
O -0.544150 3.133865 1.089958
O -0.973646 3.806087 -1.002837
H -5.043361 2.245382 2.847970
H -5.393578 3.339531 1.490479
H -3.065626 3.608524 2.478608
H -2.695924 1.965513 2.035257
H -3.255860 4.299188 0.114253
H -3.187689 2.651568 -0.437078
H 0.002384 3.761986 -1.095814

IVb
E = -5344.655858
Na -2.050430 1.040745 2.040074
I -3.018578 1.552096 -0.949516
Na -0.694419 0.315254 -2.647713
I -1.053045 -2.638038 -2.150276
Na -0.348373 -3.164903 0.724586
I -2.566858 -1.919541 2.275738
Na -3.438784 -1.495020 -0.594397
I 0.220297 2.996236 2.244070
Na -0.673872 3.567846 -0.585812
I 1.534065 2.269755 -2.221627
Na 2.547669 1.937528 0.632299
Br 2.568680 -2.735367 1.060468
C 3.348238 -2.818182 -0.783196
C 4.098750 -1.562482 -1.172645
C 5.329297 -1.230551 -0.323385
C 5.068635 -0.355350 0.876657
O 4.225020 0.519671 0.936396
O 5.917325 -0.593532 1.870604
H 2.495233 -2.998797 -1.430052
H 3.983767 -3.698348 -0.745117
H 4.429214 -1.739921 -2.201383
H 3.425195 -0.706013 -1.216898
H 5.861268 -2.126071 -0.001557
H 6.039795 -0.659245 -0.930512
H 5.752108 0.033675 2.594486

IVc
E = -5344.651093
C 5.618067 2.376017 0.593814
Br 5.318547 0.815907 1.796010
C 4.379220 2.717297 -0.207940
C 3.200830 3.233163 0.644716

C 1.866085 2.811512 0.093087
O 1.053794 3.813199 -0.176680
O 1.559964 1.634880 -0.081546
Na 0.117603 0.414454 -1.656198
I 2.592358 -1.248019 -2.274297
Na 0.838992 -3.235875 -0.891227
I 0.882202 -2.264681 2.017431
Na -2.046935 -1.785235 1.577884
I -1.971469 -2.037015 -1.540590
Na -3.941060 0.180481 -1.142258
I -2.123832 2.726847 -1.355824
Na -3.303337 2.883794 1.392583
Na 2.797507 -0.354075 0.607077
H 3.257578 2.799131 1.647636
H 4.664387 3.486706 -0.931639
H 4.081748 1.847351 -0.796191
H 6.443052 2.071774 -0.042126
H 5.937110 3.171804 1.261227
H 3.231308 4.315240 0.751389
H 0.186679 3.485848 -0.516190
I -4.366745 0.194828 1.876353

Va
E = -5085.548879
Na 0.838497 1.645541 -0.300704
I -0.020206 -1.183370 -1.961827
Na -2.609042 0.279334 -1.414489
I -1.921988 2.925269 -0.228133
Na -2.119679 1.015177 2.058253
I 0.646028 -0.097169 2.554502
Na 3.738999 -0.256440 1.619309
I 3.793265 1.605567 -0.747704
Na 3.210952 -1.099361 -1.933129
Br 3.579934 -2.730669 0.313713
O -3.644701 -0.298125 0.888702
C -4.728632 -0.643299 0.449324
O -4.854004 -0.709977 -0.883262
C -5.943305 -0.972268 1.260214
C -6.144562 -1.145723 -1.435165
C -6.580524 -2.425591 -0.753646
C -6.677908 -2.244426 0.775015
H -6.256661 -3.114931 1.276373
H -7.717260 -2.175118 1.093139
H -7.537320 -2.736034 -1.175523
H -5.860227 -3.207450 -1.001783
H -6.854832 -0.328152 -1.301922
H -5.955877 -1.276883 -2.496982
H -5.656374 -1.036779 2.307078
H -6.608433 -0.106315 1.164251
Na 0.871384 -2.498615 0.665763

Vb
E = -5085.541630
O -6.321395 -0.125627 0.903326
C -5.782800 -1.163385 0.266270
C -6.689771 -2.328311 -0.015102
C -8.058966 -1.902394 -0.590458
C -8.543658 -0.565099 0.008620
O -4.604185 -1.122363 -0.035919
Na -2.771667 0.114785 0.087325
I -0.826823 0.597305 2.421107
Na -0.346461 2.948810 0.622148
I 2.505614 2.355765 -0.579751
Na 1.514346 -0.521479 0.919153
I 4.445159 -1.705380 1.095675
Na 5.006527 1.088378 0.360386
Br -1.843687 2.195003 -1.604882
Na 0.452641 1.013975 -2.553345
I 0.637981 -1.918996 -1.772238
Na 3.241653 -3.144508 -1.196729
H -6.838004 -2.837231 0.943594
H -8.775678 -2.699508 -0.394239
H -7.981258 -1.814476 -1.673883
H -9.599522 -0.611690 0.279523
H -8.440886 0.239317 -0.722159
H -6.165515 -3.022950 -0.666628
C -7.737097 -0.192141 1.237486
H -7.977117 0.800551 1.608920
H -7.864369 -0.907541 2.053261

Vc
E = -5085.531531
Na -3.384948 3.383072 -0.853125
Na -2.747638 0.912270 1.476708
I -4.034670 0.547323 -1.296641
Na 0.501897 0.284305 2.716968
I -1.560513 -1.925502 1.949185
Na -2.032945 -1.719806 -1.191415
I 0.703235 -2.556499 -2.162999
Na 1.091469 -3.002110 0.807392
I 3.057019 -0.787879 1.540816
Na 2.826033 -0.543945 -1.475426
Br 3.414435 2.270191 -2.079765
C 3.093346 3.077439 -0.282668
C 1.625127 3.380403 -0.070641
C 1.363443 3.933307 1.337001
C -0.113351 4.261520 1.579838
C -1.053086 3.065588 1.526910
O -0.726668 1.997005 2.114739
O -2.153312 3.181840 0.913512
H -0.223361 4.695660 2.578471
H -0.463900 5.012776 0.871419
H 1.947636 4.844093 1.490204
H 1.706520 3.216361 2.086394
H 1.043271 2.467086 -0.218953
H 1.287906 4.099945 -0.821044

H 3.483147 2.345656 0.419187
H 3.719162 3.965272 -0.283798

Vd
E = -5085.529285
C 4.134324 2.597827 -0.384915
C 3.358849 3.796540 -0.893932
C 1.843489 3.614490 -1.030145
C 1.434172 2.616524 -2.112774
C -0.031572 2.205341 -2.111072
O -0.344592 1.147182 -2.727784
Br 3.641951 2.128773 1.503624
O -0.886509 2.904573 -1.502739
Na 2.587866 -0.621763 1.475485
I 2.687536 -1.900130 -1.254862
Na 0.353864 -0.931197 -2.909527
I -2.117654 -2.103615 -1.417926
Na 0.301760 -3.253438 0.101885
I 0.130777 -1.788443 2.746453
Na -2.451541 -0.851672 1.471474
I -3.817539 1.763706 0.830534
Na -2.498536 1.029642 -1.854441
H 2.017252 1.695486 -2.046193
H 1.644996 3.025842 -3.106544
H 1.439799 3.297012 -0.065128
H 1.397738 4.586839 -1.247625
H 3.963811 1.682355 -0.942898
H 5.199477 2.793990 -0.319112
H 3.792642 4.037030 -1.872792
H 3.568970 4.654272 -0.252152
Na -1.919333 3.885813 0.110057

VIa
E = -5383.958947
Na -1.015710 3.346537 0.346405
I -2.853013 1.814818 2.145093
I -1.539082 2.113086 -2.356895
Na -3.729719 0.900574 -0.603552
Na -0.845583 -0.811850 -2.563859
I -2.954535 -2.085834 -0.608531
I 1.548042 -2.571787 -2.145306
Na -1.859528 -1.032973 2.188161
Na -0.411459 -3.793310 -0.171907
I 0.625886 -2.713455 2.462513
Na 2.787242 -1.870218 0.524031
C 2.784150 3.333153 -0.670397
C 3.929619 2.641367 0.039847
C 4.744777 1.780936 -0.936381
C 5.958320 1.121818 -0.281560
C 5.636671 0.027090 0.700817
H 4.109750 1.013482 -1.384755
H 5.108201 2.403068 -1.757468
H 3.537125 2.020943 0.848324
H 4.577310 3.391612 0.499688
H 6.586371 0.651497 -1.046350
H 6.598221 1.852258 0.216360
O 6.635204 -0.199761 1.548466
O 4.604352 -0.619690 0.725572
H 6.404570 -0.939561 2.134677
H 2.078537 2.643842 -1.125912
Br 1.703515 4.411320 0.613475
H 3.114786 4.057842 -1.408791

VIb
E = -5383.957795
I 1.728861 2.256857 2.580256
Na -0.496974 3.332662 0.833481
I 0.754791 3.156712 -1.931636
Na 3.080449 2.448922 -0.122499
I 3.559787 -0.542205 -0.804722
Na 1.073378 0.247559 -2.556860
Na 2.221773 -0.667232 2.079854
I -0.567434 -2.288891 -2.444057
Na 1.930493 -3.153247 -0.889983
I 0.839570 -3.347483 1.927067
Na -1.627328 -2.913235 0.303864
Br -4.256702 -1.779953 0.937178
C -4.924997 -0.892484 -0.731298
C -5.930557 0.197018 -0.418731
C -5.424302 1.396139 0.387034
C -4.360303 2.230347 -0.349418
C -3.860949 3.384904 0.475070
O -4.817779 4.257041 0.779669
O -2.710197 3.532579 0.845338
H -4.027874 -0.544436 -1.233940
H -5.367793 -1.705804 -1.296668
H -6.783585 -0.243559 0.100697
H -6.310139 0.539904 -1.389419
H -6.275352 2.036872 0.618980
H -5.023676 1.050684 1.343126
H -4.788846 2.640364 -1.268532
H -3.491402 1.632607 -0.619412
H -4.441482 4.974993 1.315481

VIc
E = -5383.957542
C 5.527978 2.009475 0.185098
Br 5.836185 0.152639 -0.494302
C 5.198159 2.994496 -0.919745
C 3.957339 2.678671 -1.758253
C 2.637147 2.703870 -0.960544
C 1.538085 1.881661 -1.568392
O 1.901792 0.596908 -1.760035
O 0.423543 2.267632 -1.839971
Na 3.103912 -1.026780 -0.390022
Na -1.785074 2.599366 -1.618635

Na -2.119233 -2.074534 -1.456673
Na -0.886767 0.647881 2.547610
I -3.591543 0.319104 -2.464183
I 1.942848 -0.444152 2.372664
Na 0.285591 -2.824670 1.480707
I -2.612022 -1.855623 1.652716
I -2.440430 2.893844 1.335120
Na -4.206799 0.591184 0.484753
H 2.784875 2.287119 0.041870
H 3.890275 3.402404 -2.571983
H 4.092266 1.703361 -2.225822
H 6.056580 3.085441 -1.587548
H 5.086155 3.969870 -0.430328
H 2.265276 3.718753 -0.833520
H 6.463105 2.252118 0.679499
H 4.748822 1.895010 0.933062
H 1.157018 0.069438 -2.110361
I 0.835605 -2.824270 -1.522512

VId
E = -5383.954486
C -3.609910 3.667085 -0.994815
Br -2.229915 4.019154 0.406256
C -4.895263 3.111858 -0.412270
C -4.794153 1.741206 0.261458
C -4.485751 0.600627 -0.707951
C -4.085942 -0.678670 -0.032185
O -4.028893 -1.728942 -0.878441
O -3.795378 -0.799559 1.138670
Na -2.177598 -1.843530 2.351013
Na -2.048696 -2.318137 -2.376302
Na 0.021001 2.051612 0.286540
I -1.572332 -3.956644 0.137681
Na 0.946555 -2.117641 0.101564
I -0.244170 0.040027 -2.204753
Na 2.941085 0.616737 -2.040362
I 3.864470 -1.704159 -0.362065
Na 3.373790 0.476237 1.679597
I 0.329710 -0.158003 2.505805
H -5.300701 3.830020 0.302263
H -5.736994 1.530687 0.769343
H -4.031887 1.774508 1.040546
H -5.327162 0.389334 -1.372719
H -3.645259 0.848439 -1.367597
H -5.612601 3.071960 -1.241633
H -3.757396 4.640774 -1.450825
H -3.118789 3.008720 -1.705393
H -3.713333 -2.513225 -0.368951
I 2.876925 2.888320 -0.069381

Br(CH2)3COO-, LM1
E = -2880.778358
C 0.358046 0.003150 -0.477459
O -2.174569 -1.309724 0.395235
C -2.742248 -0.255138 0.020593
C -1.865891 1.046227 0.018899
C -0.409928 0.884576 0.478762
O -3.918912 -0.083956 -0.355351
Br 2.312493 -0.109414 -0.009140
H -1.904497 1.471446 -0.990528
H -2.365351 1.774016 0.666090
H -0.394932 0.426121 1.468907
H 0.075853 1.863696 0.550498
H 0.365903 0.387726 -1.494569
H -0.006239 -1.016963 -0.444322

Br(CH2)3COO-, TS
E = -2880.771686
C 0.026281 -0.432988 0.025885
O -2.011319 -1.219032 0.097586
C -2.749310 -0.186589 -0.038306
C -1.924206 1.102898 -0.217406
C -0.531602 0.918110 0.390080
O -3.983110 -0.139400 -0.062488
Br 2.387468 -0.044026 -0.041283
H -1.842324 1.289440 -1.294801
H -2.446569 1.960107 0.211946
H -0.581061 0.995645 1.478751
H 0.144322 1.699877 0.044682
H 0.019334 -0.749727 -1.000512
H 0.173355 -1.195564 0.762531

Br(CH2)3COO-, LM2
E = -2880.809565
C -1.090814 -1.419490 -0.333195
O -2.222548 -0.649237 -0.834136
C -2.361923 0.494438 -0.119527
C -1.301735 0.537315 0.960677
C -0.857114 -0.918171 1.089371
O -3.225475 1.296064 -0.366264
Br 2.350716 0.190649 -0.121237
H -0.456402 1.133061 0.595543
H -1.693431 0.998140 1.865723
H -1.481773 -1.462427 1.804424
H 0.195915 -1.000917 1.356735
H -0.218950 -1.208625 -0.954813
H -1.366708 -2.471116 -0.405076

Br(CH2)3COO.Na, LM1
E = -3043.090958
c -0.844624 1.429155 0.535002
o 1.667392 -0.459351 1.177434
br -1.644881 -0.307663 -0.083752
c 0.228762 1.992295 -0.389586
c 1.672022 1.601170 -0.024868
c 1.805013 0.084201 0.043656

o 1.917119 -0.547673 -1.042065
na 0.862078 -2.173199 0.031865
h 2.344659 1.997583 -0.786187
h 1.937855 2.029973 0.942395
h 0.139983 3.082941 -0.338635
h 0.016320 1.715066 -1.423045
h -0.471916 1.198199 1.527992
h -1.722055 2.064884 0.590120

Br(CH2)3COO.Na, TS
E = -3043.043833
c -0.423768 -1.757677 0.679218
o -1.035844 0.446146 1.098122
br 1.876177 -0.195455 -0.147818
c -1.362700 -1.764540 -0.451360
c -2.476774 -0.720475 -0.344245
c -1.795309 0.579038 0.082797
o -1.903987 1.629296 -0.574231
na 0.295308 2.014638 0.148748
h -3.008930 -0.598548 -1.284906
h -3.194221 -1.002450 0.429902
h -1.779970 -2.788841 -0.450340
h -0.813324 -1.666877 -1.388344
h -0.789614 -1.667662 1.688829
h 0.541427 -2.217344 0.552670

Br(CH2)3COO.Na, LM2
E = -3043.118364
c -3.554620 -0.577861 0.239437
o -2.910829 0.718506 0.381266
br 2.457604 -0.525616 0.082534
c -2.414674 -1.570451 -0.001826
c -1.340037 -0.688648 -0.639931
c -1.672550 0.690043 -0.121641
o -0.975754 1.683926 -0.124926
na 1.258061 1.755477 -0.047862
h -0.306949 -0.950550 -0.398675
h -1.420989 -0.657461 -1.731128
h -2.723560 -2.403840 -0.629946
h -2.055249 -1.975831 0.944598
h -4.243180 -0.510366 -0.604873
h -4.120926 -0.753613 1.150852

Br(CH2)COOH.Br(CH2)3COO-, LM1
E = -5683.537609
C -3.406961 0.397762 -0.274462
O -0.360046 0.140043 -0.088166
Br -4.965671 -0.843171 -0.412197
C -2.880708 0.490792 1.141340
C -1.772987 1.542958 1.259779
C -0.460449 1.220515 0.513949
O 0.422211 2.130614 0.608967
O 2.490718 2.168272 -0.815497
C 3.325518 1.151528 -0.771120
C 2.870288 0.063777 0.200617
Br 4.072818 -1.486780 0.284001
O 4.341836 1.098727 -1.423524
H -2.662102 -0.002287 -0.952321
H -2.122399 2.520224 0.911799
H -3.700133 0.745327 1.819586
H -2.490180 -0.482869 1.440247
H -3.793310 1.345581 -0.641410
H -1.516766 1.682074 2.314059
H 1.626633 2.072732 -0.222440
H 2.822419 0.463445 1.210014
H 1.879758 -0.301156 -0.067553

Br(CH2)COOH.Br(CH2)3COO-, TS
E = -5683.516779
C -2.499469 0.065003 -0.157780
O -0.575712 0.625057 -0.276448
Br -4.828268 -0.984772 -0.354863
C -2.731966 0.924734 1.056577
C -1.822483 2.153418 0.980038
C -0.498491 1.715654 0.372329
O 0.528738 2.408313 0.514000
O 2.706489 2.153208 -0.864491
C 3.389338 1.022108 -0.754919
C 2.714779 0.013703 0.172335
Br 3.710542 -1.657963 0.381066
O 4.429761 0.833874 -1.330864
H -2.181314 -0.954832 -0.084738
H -2.247574 2.912380 0.314568
H -3.779367 1.211176 1.107485
H -2.511490 0.341509 1.952341
H -2.689043 0.452087 -1.141515
H -1.666705 2.631898 1.947085
H 1.841095 2.176282 -0.331024
H 2.605752 0.435822 1.167736
H 1.724609 -0.241899 -0.198105

Br(CH2)COOH.Br(CH2)3COO-, LM2
E = -5683.538979
C -1.712967 -1.329123 -0.562795
O -0.654094 -0.340272 -0.317127
Br -5.079283 0.193064 -0.471894
C -2.310639 -1.610679 0.812027
C -2.107557 -0.262039 1.504679
C -0.874837 0.283323 0.845861
O -0.124208 1.154320 1.247868
O 1.959122 2.362902 0.066587
C 3.016693 1.594882 -0.192344
C 2.780878 0.126080 0.151108
Br 4.325893 -1.020135 -0.184715
O 4.034814 2.039010 -0.647312
H -1.234736 -2.180310 -1.043345

H -2.954945 0.388772 1.235770
H -3.368134 -1.855710 0.723878
H -1.769305 -2.411777 1.323142
H -2.456424 -0.869408 -1.214871
H -1.998332 -0.285282 2.586965
H 1.184429 1.867396 0.448497
H 2.535852 0.021860 1.204764
H 1.955730 -0.270371 -0.434813

Br(CH2)4COO-, LM1
E = -2920.075908
O -2.115936 -0.975195 1.063993
C 0.529869 -0.062338 0.308183
C 0.077088 1.334953 -0.056330
C -1.384017 1.638455 0.326270
C -2.447531 0.861210 -0.449072
O -3.478404 -1.264993 -0.714018
Br 2.501451 -0.280485 -0.107392
H 0.452702 -0.282506 1.365194
H 0.051341 -0.852828 -0.252771
H 0.731600 2.054545 0.445007
H 0.210909 1.487517 -1.132159
H -1.523486 1.439960 1.391710
H -1.526666 2.716130 0.172015
H -3.409882 1.376774 -0.360262
H -2.218888 0.847127 -1.520636
C -2.702690 -0.613651 0.012752

Br(CH2)4COO-, TS
E = -2920.066826
O -1.724432 -1.178201 0.292810
C 0.240226 -0.302815 0.115625
C -0.125701 1.119983 -0.188835
C -1.472514 1.581839 0.391761
C -2.695349 0.938947 -0.250726
O -3.915287 -1.108368 -0.162923
Br 2.698299 -0.176282 -0.036171
H 0.327440 -0.627616 1.134185
H 0.279973 -1.055324 -0.642555
H 0.656813 1.759545 0.219125
H -0.119083 1.272453 -1.271517
H -1.481886 1.388209 1.469323
H -1.523107 2.670341 0.270468
H -3.616346 1.394173 0.121321
H -2.683647 1.113240 -1.333654
C -2.807138 -0.580057 -0.024458

Br(CH2)4COO-, TS, decarboxylation
E = -2920.012087
C -0.097531 0.201291 -0.162120
C -2.154537 0.981326 -0.554209
C -1.439885 2.053832 0.244589
C 0.018934 1.697722 -0.036366
C -2.535094 -1.055029 0.102545
O -2.649915 -1.002792 1.287724
O -2.646106 -1.660071 -0.916399
Br 2.493650 -0.353246 0.022551
H -0.210146 -0.388533 0.729581
H 0.006752 -0.320850 -1.093217
H 0.729402 1.987407 0.736077
H 0.362440 2.125078 -0.980332
H -1.648214 1.904723 1.309218
H -1.712102 3.094117 0.007190
H -3.238355 0.966213 -0.410726
H -1.950669 1.023500 -1.624310

Br(CH2)4COO-, LM2
E = -2920.104166
O -2.040812 -1.189749 -0.249870
C -0.724314 -0.961751 -0.849747
C -0.528578 0.447690 -1.356592
C -0.810975 1.416622 -0.216359
C -2.236694 1.211049 0.293869
O -3.733505 -0.517840 0.982605
Br 2.734262 -0.084795 0.264613
H 0.039698 -1.185795 -0.102454
H -0.666007 -1.702944 -1.645864
H 0.514900 0.532759 -1.666389
H -1.181034 0.646869 -2.216303
H -0.073017 1.227531 0.567425
H -0.674971 2.453555 -0.533582
H -2.400530 1.660011 1.274615
H -2.955053 1.692170 -0.380634
C -2.717541 -0.229548 0.392138

Br(CH2)4COO.Na, LM1
E = -3082.393305
c -0.898720 1.089160 0.956378
o 1.589871 -0.771927 1.238218
br -1.766594 -0.310641 -0.200847
na 0.541913 -2.359808 0.076285
o 1.953128 -1.003893 -0.950578
c 1.910176 -0.310944 0.102062
c 2.145063 1.191655 -0.044140
c 0.946591 1.879738 -0.729036
c -0.252295 2.210855 0.170147
h -0.190135 0.521184 1.550442
h -1.727621 1.444453 1.560967
h -1.011852 2.726945 -0.423081
h 0.071495 2.927182 0.938092
h 1.283723 2.826389 -1.159485
h 0.624894 1.260898 -1.569615
h 2.344401 1.636938 0.932611
h 3.025951 1.330111 -0.672998

Br(CH2)4COO.Na, TS

E = -3082.339751
c -0.212372 -1.554944 1.212944
o -0.939140 0.727955 1.167998
br 1.925935 -0.211868 -0.237580
na 0.578372 2.123955 0.206154
o -1.605926 2.006180 -0.513589
c -1.668504 0.947083 0.141699
c -2.544193 -0.199337 -0.342405
c -1.672838 -1.316685 -0.929487
c -0.902118 -2.199573 0.089626
h -0.787686 -1.146785 2.023404
h 0.819373 -1.787794 1.414639
h -0.202396 -2.821239 -0.468144
h -1.648921 -2.851219 0.574944
h -2.292138 -1.992141 -1.522006
h -0.931751 -0.883766 -1.603672
h -3.159058 -0.576716 0.479425
h -3.206550 0.179184 -1.120496

Br(CH2)4COO.Na, LM2
E = -3082.413617
c -1.348795 -0.974066 1.325862
o -1.048844 0.421699 1.022113
br 2.392888 -0.404505 -0.186813
na 0.984281 1.730889 0.230260
o -1.267070 2.081759 -0.387635
c -1.687340 1.008330 -0.001372
c -2.855302 0.259971 -0.571193
c -2.491345 -1.207173 -0.913517
c -1.417673 -1.769685 0.037801
h -2.279022 -1.002564 1.899377
h -0.525151 -1.293863 1.958219
h -0.427486 -1.722172 -0.420929
h -1.613513 -2.816291 0.277486
h -3.404538 -1.801990 -0.871844
h -2.130199 -1.255795 -1.940557
h -3.637136 0.272275 0.195072
h -3.231100 0.806388 -1.432566

Br(CH2)COOH.Br(CH2)4COO-, LM1
E = -5722.834421
C 2.690894 -0.116410 0.251202
O 0.108018 1.369118 0.988060
Br 3.823422 -1.682845 -0.271340
C 3.518740 1.151746 0.278591
C 2.745843 2.390940 0.763800
C 1.612071 2.865090 -0.145544
C 0.254480 2.153137 0.035896
O -0.617305 2.470415 -0.833498
O -3.082782 2.159695 -0.479709
C -3.575396 0.964935 -0.228889
O -4.749671 0.761880 -0.026132
C -2.504269 -0.124232 -0.242655
Br -3.180589 -1.913880 0.199653
H -1.705458 0.109962 0.459995
H -2.070630 -0.203058 -1.236267
H -2.037948 2.210050 -0.591308
H 1.893121 2.799059 -1.201273
H 1.414395 3.926083 0.036154
H 3.481100 3.196112 0.869692
H 2.334784 2.197467 1.757173
H 3.920674 1.340348 -0.721749
H 4.378594 0.993050 0.935322
H 2.278509 -0.378741 1.218500
H 1.893432 -0.105071 -0.481391

Br(CH2)COOH.Br(CH2)4COO-, TS
E = -5722.811009
C 2.359468 0.064955 0.066501
O 0.545059 0.926470 0.203011
Br 4.319596 -1.627913 0.423735
C 2.996158 0.968123 -0.942662
C 2.764713 2.455276 -0.650954
C 1.334064 2.910128 -0.913645
C 0.263801 2.099813 -0.192602
O -0.859351 2.630857 -0.053750
O -2.801268 1.780061 1.429347
C -3.393563 0.647848 1.076951
O -4.348567 0.204550 1.660870
C -2.733055 -0.009369 -0.133206
Br -3.610910 -1.657940 -0.716125
H -1.696450 -0.253044 0.087198
H -2.748414 0.668230 -0.982390
H -2.010708 2.037649 0.843613
H 1.110298 2.834978 -1.984140
H 1.189413 3.958134 -0.646394
H 3.447100 3.044022 -1.270540
H 3.037209 2.661764 0.389127
H 2.629407 0.716020 -1.941665
H 4.065098 0.764428 -0.938816
H 2.497471 0.243232 1.115672
H 1.839064 -0.826681 -0.216108

Br(CH2)COOH.Br(CH2)4COO-, LM2
E = -5722.833183
C 1.672248 -0.912390 0.050043
O 0.549695 0.037177 -0.086236
Br 5.102956 -0.250186 0.984206
C 2.340273 -1.213047 -1.269523
C 2.780829 0.100091 -1.905312
C 1.574781 1.018131 -2.099153
C 0.533533 0.988259 -1.002072
O -0.378924 1.805269 -0.987106
O -2.358441 2.265596 0.755282
C -3.311229 1.336468 0.822120
O -4.299839 1.478592 1.487618

C -2.997920 0.105974 -0.026012
Br -4.396395 -1.255777 0.012626
H -2.082070 -0.365570 0.320757
H -2.865033 0.391101 -1.066192
H -1.603267 2.023739 0.152536
H 1.018142 0.747051 -3.004185
H 1.859948 2.063211 -2.228895
H 3.269877 -0.070222 -2.866877
H 3.522767 0.550733 -1.240175
H 1.668933 -1.776193 -1.929592
H 3.215495 -1.826041 -1.047622
H 2.399571 -0.476188 0.739482
H 1.210983 -1.786907 0.504628

Br(CH2)7COO-, LM1
E = -3037.974714
O 1.438084 -1.446841 -1.248815
C -1.256734 -0.000371 -0.173283
Br -3.238578 -0.181315 0.091414
C -0.879702 1.461278 -0.324287
C 0.631523 1.650722 -0.548424
C 1.473445 1.469040 0.718184
C 2.990045 1.500904 0.482421
C 3.573068 0.355183 -0.363016
C 3.305827 -1.052178 0.183211
C 1.852066 -1.564517 -0.068342
O 1.239488 -2.025935 0.921860
H 3.524808 -1.103644 1.253915
H 3.977407 -1.754953 -0.322839
H 3.177966 0.393396 -1.381186
H 4.654774 0.525578 -0.440648
H 3.484987 1.492857 1.461501
H 3.260805 2.457489 0.014488
H 1.213719 0.526188 1.202680
H 1.214604 2.264475 1.429538
H 0.962036 0.939617 -1.307690
H 0.796564 2.659934 -0.946723
H -1.201381 2.021201 0.560206
H -1.426758 1.878659 -1.174507
H -0.816288 -0.501434 0.682658
H -1.030835 -0.591500 -1.054043

Br(CH2)7COO-, TS
E = -3037.952604
O 1.015221 -0.898976 -0.948030
C -0.878249 -0.197195 -0.374101
Br -3.386200 -0.089452 0.067816
C -0.617794 1.282593 -0.181320
C 0.830885 1.788502 -0.337240
C 1.760217 1.421542 0.831639
C 3.246859 1.287004 0.465837
C 3.607383 0.173913 -0.534156
C 3.211400 -1.243891 -0.108650
C 1.685008 -1.478044 -0.021746
O 1.239007 -2.156004 0.907858
H 3.645008 -1.505241 0.860091
H 3.608568 -1.957733 -0.838829
H 3.144597 0.380471 -1.500838
H 4.692018 0.208592 -0.694500
H 3.812977 1.121680 1.390809
H 3.605202 2.242028 0.060062
H 1.423574 0.494907 1.296661
H 1.667229 2.186384 1.611739
H 1.235997 1.413360 -1.274077
H 0.780503 2.880635 -0.423253
H -0.983314 1.557709 0.810058
H -1.248308 1.816225 -0.893450
H -0.812764 -0.898874 0.435774
H -1.162344 -0.576035 -1.334002

Br(CH2)7COO-, LM2
E = -3037.986867
O 0.982735 -1.057939 -0.526883
C -0.153016 -1.192821 0.379088
Br -3.596261 0.065335 -0.292654
C -0.214334 -0.026621 1.367080
C 0.343920 1.300295 0.815893
C 1.871558 1.485389 1.021516
C 2.719901 1.858758 -0.206895
C 2.794512 0.843551 -1.360507
C 3.229679 -0.578291 -0.970498
C 2.217775 -1.311579 -0.102842
O 2.505322 -2.010323 0.842393
H 4.180927 -0.584602 -0.435087
H 3.369534 -1.170368 -1.880987
H 1.829639 0.780267 -1.863521
H 3.505225 1.227763 -2.099910
H 3.740660 2.058124 0.143134
H 2.354565 2.805460 -0.620482
H 2.292326 0.587239 1.479694
H 2.033013 2.272457 1.765530
H 0.074815 1.386402 -0.238315
H -0.180523 2.117750 1.314270
H 0.305244 -0.277793 2.297302
H -1.277304 0.096467 1.589262
H -0.097314 -2.157472 0.882135
H -1.026114 -1.154395 -0.271239

Br(CH2)7COO.Na, LM1
E = -3200.292668
c -1.268265 -1.026571 0.752476
o 0.850890 1.341985 1.031482
br -2.764472 0.058136 -0.044585
c 1.667323 1.578418 0.088725
o 1.356535 2.215982 -0.954566
c 3.100050 1.071887 0.244038

c 3.241135 -0.396486 0.676248
c 2.991308 -1.426717 -0.437084
c 1.530008 -1.594408 -0.874275
c 0.670657 -2.454081 0.065692
c -0.837951 -2.194147 -0.122789
na -0.751142 2.270826 -0.235151
h -0.481345 -0.291790 0.904079
h -1.669807 -1.338004 1.711954
h -1.030363 -1.977365 -1.175137
h -1.429405 -3.077241 0.126561
h 0.883271 -3.511207 -0.115030
h 0.943251 -2.267343 1.109074
h 1.500895 -2.051164 -1.868569
h 1.075268 -0.609408 -0.995118
h 3.589651 -1.141850 -1.308329
h 3.376995 -2.400341 -0.114180
h 2.579719 -0.576024 1.527540
h 4.260823 -0.543012 1.043907
h 3.556254 1.702965 1.013465
h 3.638876 1.256830 -0.686592

Br(CH2)7COO.Na, TS
E = -3200.251104
c -0.590316 -0.552318 0.676707
o 1.143226 0.589279 1.308604
br -3.016436 -0.318412 -0.171786
c 1.410972 1.403121 0.378573
o 0.555001 2.100737 -0.232974
c 2.875833 1.506861 -0.047113
c 3.679215 0.210759 0.121753
c 3.366513 -0.914270 -0.881899
c 1.915143 -1.417147 -0.943675
c 1.400532 -2.089425 0.341014
c -0.129176 -1.963941 0.564029
na -1.627921 2.084039 -0.279147
h -0.437013 0.089395 -0.159885
h -1.000978 -0.156616 1.582203
h -0.644216 -2.405551 -0.293651
h -0.421727 -2.531325 1.447602
h 1.631199 -3.157619 0.314497
h 1.902771 -1.673029 1.210494
h 1.831391 -2.124432 -1.774467
h 1.272110 -0.579793 -1.221899
h 3.645053 -0.573271 -1.884936
h 4.027809 -1.758879 -0.659521
h 3.542902 -0.145565 1.143812
h 4.741693 0.450924 0.020104
h 3.326529 2.289219 0.572522
h 2.906765 1.874562 -1.075131

Br(CH2)7COO.Na, LM2
E = -3200.299922
c 0.325906 -0.714886 1.530943
o 1.455558 0.200112 1.384345
br -3.227349 -0.315267 -0.299785
c 1.343823 1.269233 0.611959
o 0.287258 1.822469 0.351706
c 2.671750 1.720357 0.057316
c 3.565994 0.572178 -0.440666
c 2.984338 -0.318809 -1.552610
c 1.729915 -1.153035 -1.223836
c 1.881500 -2.107041 -0.016467
c 0.729698 -2.101598 1.004309
na -1.861937 1.858989 -0.142443
h -0.528268 -0.320756 0.986692
h 0.077594 -0.738406 2.590210
h -0.168633 -2.543340 0.566002
h 1.018861 -2.742706 1.840001
h 1.997109 -3.132606 -0.373976
h 2.805422 -1.877404 0.515881
h 1.479336 -1.731594 -2.115921
h 0.869950 -0.494731 -1.085854
h 2.765976 0.301859 -2.428282
h 3.781191 -1.001803 -1.864345
h 3.857859 -0.037994 0.414843
h 4.488018 1.022603 -0.817492
h 3.195581 2.262931 0.850892
h 2.458442 2.440369 -0.733422

Br(CH2)COOH.Br(CH2)7COO-, LM1
E = -5840.730557
C 5.277169 -0.259540 -0.329470
O -1.422245 0.656836 -0.037170
Br 7.030581 -1.106253 0.073145
H 5.507636 0.795444 -0.435855
H 4.989904 -0.669459 -1.294324
C 4.246854 -0.540293 0.745265
H 4.142143 -1.621620 0.860449
H 4.606079 -0.153482 1.702998
C 2.875715 0.072561 0.414743
H 2.152409 -0.304227 1.143196
H 2.537428 -0.301282 -0.555631
C 2.835782 1.604208 0.434100
H 3.251678 1.947832 1.388965
H 3.490601 2.009853 -0.346596
C 1.430283 2.191854 0.262565
H 1.473369 3.266863 0.472520
H 0.761034 1.753032 1.007082
C 0.815506 1.986752 -1.123846
H 1.468867 2.448315 -1.876058
H 0.762628 0.919884 -1.348921
C -0.592958 2.563249 -1.245483
H -0.910470 2.560452 -2.293703
H -0.622717 3.609604 -0.927939
C -1.678424 1.793929 -0.462122
O -2.783265 2.418540 -0.351718

O -4.620552 1.639219 1.152760
C -5.136486 0.442072 0.985571
O -6.024554 -0.003809 1.675517
C -4.497693 -0.310079 -0.181583
H -3.421510 -0.398472 -0.038078
H -4.673980 0.225982 -1.110282
H -3.820048 1.892836 0.503784
Br -5.211855 -2.120493 -0.455007

Br(CH2)COOH.Br(CH2)7COO-, TS
E = -5840.685345
C 1.888337 -0.575752 0.122153
O 0.336911 0.654521 -0.175154
Br 3.498701 -2.618121 -0.155262
H 2.319916 -0.331993 -0.825679
H 1.145099 -1.346790 0.126782
C 2.476993 -0.080233 1.422748
H 2.208822 -0.815929 2.181626
H 3.561904 -0.137288 1.327758
C 2.109740 1.318500 1.974652
H 2.332270 1.260954 3.044938
H 1.037630 1.491868 1.891156
C 2.889946 2.533715 1.440305
H 2.827058 3.314353 2.206565
H 3.953041 2.271819 1.369824
C 2.441773 3.168687 0.115848
H 2.965573 4.124316 0.017514
H 1.380735 3.424336 0.186560
C 2.701851 2.317634 -1.147518
H 3.352429 2.866993 -1.833263
H 3.269228 1.430080 -0.870885
C 1.440024 1.922544 -1.936990
H 1.672410 1.089338 -2.608454
H 1.114023 2.752716 -2.563309
C 0.228837 1.532640 -1.088755
O -0.846187 2.124800 -1.334303
O -2.889898 2.239196 0.245252
C -3.599285 1.136300 0.440322
O -4.585753 1.112378 1.130129
C -3.028294 -0.069211 -0.303040
H -2.009445 -0.266290 0.021808
H -3.010915 0.124374 -1.372218
H -2.077183 2.113759 -0.353918
Br -4.054779 -1.714528 -0.047953

Br(CH2)COOH.Br(CH2)7COO-, LM2
E = -5840.715959
C 1.318070 -0.173849 1.144341
O 0.265617 0.280685 0.243505
Br 4.137886 -2.147588 -0.057583
H 2.224503 -0.432432 0.601388
H 0.947182 -1.104006 1.573019
C 1.535257 0.865561 2.233906
H 0.711549 0.800561 2.952154
H 2.441475 0.567722 2.770993
C 1.641105 2.333511 1.795548
H 1.677073 2.920556 2.718717
H 0.711373 2.628296 1.299786
C 2.837396 2.765924 0.927945
H 3.000156 3.829677 1.130482
H 3.744409 2.254150 1.267170
C 2.722267 2.617660 -0.602305
H 3.442923 3.316425 -1.036670
H 1.740776 2.979350 -0.929446
C 3.023522 1.219713 -1.180024
H 3.672460 1.329521 -2.050519
H 3.610863 0.630105 -0.474012
C 1.834580 0.349580 -1.669833
H 2.134746 -0.697642 -1.533283
H 1.658817 0.521365 -2.730313
C 0.490253 0.576257 -1.032224
O -0.454568 1.018774 -1.677863
O -2.725466 2.041611 -0.745299
C -3.654203 1.197776 -0.297211
O -4.722938 1.574804 0.099507
C -3.204445 -0.260076 -0.349090
H -2.324138 -0.403783 0.272092
H -2.952769 -0.541123 -1.368420
H -1.882566 1.595528 -1.043016
Br -4.558537 -1.523086 0.268862

Br(CH2)10COO-, LM1
E = -3155.871766
O 0.944429 -1.751462 -0.882557
C -1.955759 -0.685701 -0.426178
Br -3.881555 -0.368060 0.112748
O 0.880225 -1.531116 1.358254
C -1.268529 0.575373 -0.889585
C -1.027681 1.603236 0.217366
C -0.074467 2.760278 -0.158096
C 1.357335 2.594944 0.374483
C 2.214013 1.532764 -0.327890
C 3.373961 1.055593 0.548469
C 4.372366 0.104827 -0.123544
C 3.814607 -1.217613 -0.677986
C 2.984541 -2.052653 0.310876
C 1.455613 -1.746223 0.266871
H 1.876685 3.560498 0.326585
H 3.082361 -3.110499 0.044532
H 3.352183 -1.941741 1.334641
H 4.672999 -1.799803 -1.035047
H 3.184769 -1.034771 -1.551904
H 5.153784 -0.128665 0.610896
H 4.878216 0.637206 -0.940170
H 3.929447 1.927545 0.920012
H 2.946163 0.564335 1.427298

H 2.596487 1.941929 -1.272239
H 1.603359 0.671033 -0.592370
H 1.289358 2.343307 1.438676
H -0.476421 3.695636 0.245791
H -0.053192 2.891223 -1.246980
H -0.622667 1.074639 1.085107
H -1.993062 2.012622 0.527272
H -0.315013 0.205776 -1.277606
H -1.815294 1.029910 -1.722941
H -2.034364 -1.430146 -1.209460
H -1.474591 -1.116254 0.447447

Br(CH2)10COO-, TS
E = -3155.855177
O 0.480812 -1.479442 -0.548832
C -1.451956 -0.660230 -0.350508
Br -3.949915 -0.306064 -0.009277
O 0.883446 -1.375137 1.664809
C -1.058852 0.715108 -0.796068
C -0.713407 1.658203 0.364532
C 0.212658 2.838668 -0.002032
C 1.681732 2.605112 0.385416
C 2.390625 1.488894 -0.397254
C 3.521133 0.823220 0.389001
C 4.263272 -0.289409 -0.362538
C 3.418944 -1.474047 -0.864152
C 2.604701 -2.217060 0.208765
C 1.201219 -1.631227 0.498549
H 2.245993 3.540036 0.282720
H 2.434536 -3.242830 -0.132944
H 3.154004 -2.274187 1.151405
H 4.109631 -2.178896 -1.340740
H 2.729945 -1.150510 -1.647135
H 5.049056 -0.678838 0.296333
H 4.783170 0.147203 -1.224926
H 4.253069 1.580950 0.697084
H 3.096898 0.413832 1.310491
H 2.775698 1.890275 -1.343396
H 1.669301 0.720296 -0.665156
H 1.704456 2.354332 1.451540
H -0.131598 3.742197 0.511493
H 0.138230 3.059100 -1.074033
H -0.239655 1.071493 1.155787
H -1.651649 2.030038 0.781621
H -0.203986 0.593440 -1.464171
H -1.863311 1.136799 -1.400842
H -1.729961 -1.402092 -1.072845
H -1.411275 -0.947153 0.682313

Br-
E = -2574.264150 in
Br 0.000000 0.000000 0.000000

Br(CH2)4COOH.Br-
E = -5494.918646
C -1.867759 -1.088858 -0.535791
Br -3.588697 -0.217946 0.002468
C -0.782359 -0.974407 0.508413
C -0.231741 0.426747 0.777270
C 0.336917 1.107994 -0.482195
C 1.271482 2.250494 -0.122941
O 0.924137 3.411671 -0.127349
O 2.509913 1.907286 0.228795
H -1.131255 -1.421744 1.443155
H 0.570210 0.312567 1.508111
H -0.997286 1.064930 1.226447
H 0.895244 0.365591 -1.057954
H -0.464196 1.523974 -1.093849
H 0.055675 -1.591188 0.156389
H -2.152597 -2.120346 -0.720125
H -1.611430 -0.607232 -1.474481
H 2.685722 0.922080 0.145276
Br 3.083505 -1.248685 -0.054270

Br(CH2)7COOH.Br-
C 2.012648 -0.630358 -0.577523
C 1.042478 -1.427141 0.269961
C -0.296269 -1.623482 -0.455150
C -1.348018 -2.273904 0.448918
C -2.631867 -2.726131 -0.261235
C -3.491685 -1.643902 -0.930894
C -4.131554 -0.621481 0.022726
C -3.189086 0.453732 0.551194
O -3.059391 0.696605 1.730840
Br 3.861252 -0.687406 0.172638
O -2.570230 1.072536 -0.442506
H -2.361938 -3.462887 -1.027728
H -4.297825 -2.152653 -1.470302
H -2.911601 -1.105453 -1.682406
H -4.928440 -0.097811 -0.515107
H -4.584968 -1.116270 0.883373
H -3.255902 -3.262050 0.464170
H -0.904999 -3.158368 0.922838
H -1.590704 -1.588642 1.265064
H -0.135376 -2.253017 -1.340006
H -0.653160 -0.656386 -0.813424
H 0.875837 -0.894177 1.208813
H 1.469405 -2.403345 0.518909
H 2.126746 -1.037999 -1.579233
H 1.748372 0.425857 -0.616970
H -1.800240 1.674868 -0.163030
Br 0.094230 2.684298 -0.194627

Na6I4Br+
E = -4739.691856
Na -1.035013 1.608231 1.739380

I -3.729425 0.714673 0.902443
Na -2.378564 0.627469 -1.803438
I -1.244170 -2.165160 -1.859267
Na 1.111074 -0.822692 -0.613619
Na -2.264970 -1.933836 0.969523
I 0.320344 2.304457 -0.973261
Na 3.300381 2.289022 -0.972625
I 4.134395 -0.370380 0.027099
Na 2.977227 -1.158858 2.633293
Br 0.248225 -0.923799 2.268017

Na4I2Br+
E = -3819.213097
Br -0.942544 2.596139 0.000000
Na 0.247129 1.959707 2.403988
I 0.247129 -1.020564 2.371166
Na -1.529586 -0.191274 0.000000
I 0.247129 -1.020564 -2.371166
Na 1.652912 -2.154054 -0.000000
Na 0.247129 1.959707 -2.403988

Na2Br+
E = -2898.727159 in
Na 0.000000 0.000000 2.628404
Br 0.000000 0.000000 0.000000
Na 0.000000 0.000000 -2.628404

Na4I3+
E = -1542.826285
I -1.174132 2.464488 0.081961
Na 1.637130 2.378747 -0.899836
I 2.721916 -0.215480 0.082737
Na -0.000879 -0.001015 1.509038
I -1.547306 -2.249016 0.081996
Na 1.241954 -2.605759 -0.900980
Na -2.880507 0.228064 -0.896838

Na3I2+
E = -1082.578517
I -2.121345 0.000039 -0.000000
Na 0.000417 -1.077657 -1.864033
I 2.121082 0.000035 -0.000000
Na 0.000417 -1.076513 1.864694
Na 0.000436 2.153816 -0.000660

Na2I+
E = -622.339758
Na 0.000000 2.759917 -0.498486
I 0.000000 0.000000 0.206919
Na -0.000000 -2.759917 -0.498486

Na6I5+
E = -2463.305631
Na -1.961153 1.155742 1.812029
I -0.000059 -1.241524 2.389925
Na -1.960985 -2.147363 0.094905
I -4.019869 -0.000319 0.000004
Na -1.961493 0.991134 -1.907178
I -0.000057 2.691015 -0.119621
I 0.000106 -1.448739 -2.270351
Na 1.961606 0.991300 -1.907126
I 4.019877 -0.000251 0.000114
Na 1.960937 1.155689 1.811955
Na 1.961095 -2.147383 0.095071

[Na4I2(Br(CH2)4COO)]+
E = -4165.047709
C -0.679802 -1.859337 0.358890
C -1.991578 -1.872245 1.134243
C -3.213758 -2.327093 0.327487
C -4.562131 -1.833737 0.858285
C -4.754689 -0.332289 0.922334
Br -4.511423 0.577023 -0.850251
O -0.692282 -1.539865 -0.870150
O 0.391210 -2.117573 0.974638
H -2.118991 -0.839858 1.485937
H -1.854712 -2.473123 2.033065
H -3.104121 -2.007634 -0.709208
H -3.235116 -3.418446 0.300180
H -4.719766 -2.191675 1.883660
H -5.360487 -2.272497 0.256961
H -5.768744 -0.059305 1.196084
H -4.056550 0.180450 1.576507
Na 1.526778 -2.266712 -1.072422
Na 1.716928 -0.501704 1.776417
I 3.979233 -0.684738 -0.313044
Na 3.062332 2.091059 -0.678389
I 0.357495 2.069886 0.546675
Na -1.588581 0.502914 -1.215623

[(CH2)4COO.Br(CH2)4COOH]
E = -3266.444441
C 2.101224 -1.899250 1.017535
H 1.997614 -2.948696 1.296915
C 3.539184 -1.551998 0.686996
C 3.767040 -0.068195 0.397565
C 5.240534 0.238164 0.082710
C 5.463438 1.708544 -0.161059
O 5.085572 2.078512 -1.409882
O 5.896813 2.495590 0.639470
Br -4.849820 1.462866 0.395925
C -4.225040 -0.224754 -0.421637
C -2.715879 -0.266727 -0.553784
C -2.253562 -1.564258 -1.228481
C -0.735156 -1.662484 -1.362366
C -0.023517 -1.904110 -0.047608

O 1.295008 -1.651122 -0.157209
O -0.543389 -2.297858 0.965646
H 4.153453 -1.860656 1.539009
H 5.875022 -0.050916 0.920491
H 3.144489 0.242289 -0.443066
H 3.455347 0.523690 1.263499
H 3.863768 -2.154228 -0.167036
H 5.551101 -0.321751 -0.802047
H 5.227567 3.036089 -1.476535
H -4.603674 -1.010605 0.226547
H -4.728641 -0.274188 -1.383887
H -2.378036 0.593615 -1.137803
H -2.264631 -0.183410 0.436372
H -2.614512 -2.424623 -0.660490
H -2.693608 -1.632328 -2.227420
H -0.465912 -2.498964 -2.015416
H -0.312795 -0.770877 -1.831198
H 1.715521 -1.295328 1.841271

(CH2)4OCO
E = -345.817590
C 1.730242 -0.061777 -0.319112
C 1.121273 1.196158 0.288428
C -0.350590 1.281907 -0.111080
C -1.136740 -0.015672 -0.013331
O -0.460936 -1.189493 -0.037765
O -2.336690 -0.038531 0.013651
H 1.650987 2.093072 -0.037863
H 1.213007 1.154983 1.378412
H 1.690396 -0.011289 -1.411657
H 2.780182 -0.175476 -0.038618
H -0.433362 1.585183 -1.160434
H -0.898641 2.027143 0.464438
C 0.965699 -1.274569 0.171499
H 1.129489 -1.420694 1.243820
H 1.269631 -2.185010 -0.343597

Br(CH2)4COOH
E = -2920.613493
C -1.421352 0.974281 0.752734
Br -2.339036 -0.534520 -0.142683
C -0.390366 1.648200 -0.133939
C 0.806393 0.794768 -0.560186
C 1.673703 0.315423 0.616269
C 2.897397 -0.433814 0.152042
O 3.087852 -1.615574 0.269297
O 3.795406 0.388832 -0.446501
H -0.888529 2.032857 -1.026673
H 1.425015 1.386423 -1.237230
H 0.453905 -0.070931 -1.125377
H 2.001172 1.177040 1.204985
H 1.119759 -0.360536 1.265378
H -0.029261 2.524245 0.420317
H -2.219734 1.658259 1.024583
H -0.995927 0.546468 1.656033
H 4.539152 -0.164840 -0.731996

[(CH2)7COO.Br(CH2)7COOH-]
E = -3502.245481
C 2.972148 -1.729613 0.832542
C 1.834639 -2.071174 -0.106926
C 0.489630 -2.079999 0.630548
C -0.685326 -2.439739 -0.281689
C -2.047513 -2.289328 0.396341
C -3.210933 -2.731529 -0.493433
C -4.613817 -2.488178 0.101950
C -5.201056 -1.124483 -0.191092
O -6.226782 -0.939853 -0.797502
Br 4.697530 -1.527549 -0.140494
O -4.445498 -0.120604 0.300350
O 3.891330 1.865519 -0.869515
C 2.904516 2.234617 -0.034660
O 2.546980 1.555454 0.898680
C 2.329095 3.585876 -0.395198
C 0.955708 3.842955 0.222989
C -0.150690 2.972263 -0.375734
C -1.492578 3.102245 0.344814
C -2.560349 2.159623 -0.210745
C -3.877605 2.200168 0.564462
C -4.922308 1.224940 0.058105
H 0.162224 1.924758 -0.339360
H 2.308349 3.673883 -1.483873
H 0.705066 4.899429 0.091173
H 1.016439 3.664044 1.298993
H -0.277267 3.221689 -1.435681
H 3.054392 4.329972 -0.049284
H 4.226349 0.990458 -0.589308
H 3.165608 -2.507783 1.565854
H 2.833690 -0.769014 1.318793
H 1.797145 -1.334086 -0.912578
H 2.015694 -3.042377 -0.575057
H 0.528794 -2.786572 1.466888
H 0.319917 -1.092189 1.070561
H -0.657936 -1.801386 -1.171914
H -0.562823 -3.467335 -0.642296
H -1.344884 2.888324 1.409192
H -1.846721 4.138075 0.288255
H -2.752877 2.402098 -1.262177
H -2.171807 1.137598 -0.199330
H -3.692809 2.003590 1.625135
H -4.311903 3.203804 0.506612
H -5.106606 1.343685 -1.011167
H -5.874890 1.350712 0.575356
H -2.064431 -2.880286 1.319521
H -2.189754 -1.248856 0.696570
H -3.140866 -2.239715 -1.468707

H -3.113305 -3.802550 -0.692099
H -5.326515 -3.206452 -0.299619
H -4.581432 -2.625278 1.187105

(CH2)7OCO
E = -463.706640
O -0.633642 -0.920271 -0.833006
C -1.983623 -0.480980 -0.587110
C -2.005210 0.867443 0.146367
C -0.796413 1.778334 -0.158486
C 0.419058 1.547224 0.779910
C 1.791043 1.299988 0.129296
C 1.961798 0.044386 -0.743240
C 1.565323 -1.289372 -0.087480
C 0.083270 -1.410000 0.199388
O -0.406277 -1.853746 1.206392
H 2.090788 -1.457367 0.853370
H 1.837707 -2.110324 -0.758229
H 1.401295 0.151941 -1.672340
H 3.015325 -0.021244 -1.029001
H 2.529801 1.261815 0.937293
H 2.062456 2.168422 -0.480305
H 0.199034 0.726938 1.465957
H 0.528557 2.422301 1.426120
H -0.501472 1.654935 -1.203392
H -1.120261 2.816277 -0.061083
H -2.062770 0.701009 1.224225
H -2.935032 1.368123 -0.136981
H -2.530582 -1.247367 -0.038611
H -2.406974 -0.385461 -1.586001

Br(CH2)7COOH
E = -3038.516771
C 3.233588 -0.774540 -0.000155
C 2.008570 0.117651 0.000019
C 0.713349 -0.702612 -0.000126
C -0.543588 0.168957 0.000044
C -1.842092 -0.637930 -0.000078
C -3.094689 0.238269 0.000068
C -4.381271 -0.579459 -0.000022
C -5.634268 0.260189 0.000059
O -5.686637 1.462093 0.000221
Br 4.914061 0.268253 0.000027
O -6.748219 -0.513343 -0.000107
H -4.431187 -1.241725 0.870098
H -4.431179 -1.241562 -0.870267
H -3.087376 0.897199 0.872097
H -3.087411 0.897439 -0.871778
H -1.857577 -1.297414 0.875417
H -1.857587 -1.297135 -0.875783
H -0.526791 0.827613 -0.875086
H -0.526767 0.827305 0.875404
H 0.697670 -1.360022 -0.876536
H 0.697681 -1.360348 0.876039
H 2.033774 0.769799 -0.876643
H 2.033785 0.769471 0.876924
H 3.290918 -1.400010 -0.887340
H 3.290930 -1.400338 0.886798
H -7.509769 0.087724 -0.000059

[(CH2)3COO]
E = -306.521586
C -0.888973 -0.002025 0.002756
O -2.081508 -0.030318 -0.067992
O -0.128489 -1.130713 -0.044164
C 1.269154 -0.821246 0.123539
C 1.405099 0.668894 -0.208964
C 0.023143 1.205687 0.160642
H -0.333631 2.028355 -0.454976
H -0.030461 1.527243 1.204488
H 2.221910 1.139250 0.336559
H 1.591472 0.800381 -1.276019
H 1.546736 -1.039748 1.158004
H 1.833407 -1.475091 -0.538639

[(NaI)2(CH2)3COO]
E = -1226.977243
C -5.118069 0.558477 -0.832689
C -3.604840 0.356573 -0.930978
C -3.044081 1.398934 0.009284
O -4.011592 1.889800 0.792226
C -5.273186 1.215911 0.542019
O -1.899571 1.777275 0.108938
Na 0.173682 0.931324 0.134240
I 3.134873 1.216937 -0.056855
I -0.326459 -2.089283 0.099715
Na 2.532969 -1.609942 -0.054576
H -6.055314 1.969421 0.593754
H -5.466833 1.232830 -1.615790
H -5.684789 -0.367657 -0.907685
H -5.420055 0.488295 1.341858
H -3.273906 -0.620829 -0.566917
H -3.187830 0.481083 -1.928238

[(NaI)4(CH2)3COO]
E = -2147.457760
C 4.750527 0.401222 0.005127
O 3.645298 0.880742 -0.059589
O 4.957600 -0.920002 -0.067556
C 6.363809 -1.237313 0.096262
C 7.108877 0.070338 -0.189999
C 6.067130 1.127052 0.182408
H 6.505747 -1.587884 1.120026
H 6.593932 -2.044600 -0.594527
H 8.032849 0.148098 0.380008
H 7.358227 0.138687 -1.249532

H 6.085904 2.031001 -0.422325
H 6.135754 1.431414 1.230667
Na 1.419338 0.489624 -0.107858
I 0.297594 -1.050194 2.412021
Na -1.869420 0.976469 1.826847
I 0.137880 -1.280204 -2.378581
Na -1.279299 -2.231552 0.117124
I -3.858565 -0.598181 0.125757
Na -1.995811 0.796624 -1.853223
I -0.620316 2.884933 -0.155834

[Na2I(Br(CH2)3COO)]
E = -3503.361412
Na 0.444347 -1.166956 -0.099752
Br 3.133657 -1.686303 0.079872
Na 3.079833 0.983151 0.057119
I 0.328143 1.904566 -0.101807
O -1.722234 -1.745032 -0.081629
C -2.805060 -1.212603 0.001176
O -3.824279 -1.578818 -0.784886
C -4.983544 -0.734725 -0.557609
C -4.751671 -0.083544 0.809256
C -3.225987 -0.089408 0.920960
H -2.838871 -0.255103 1.924270
H -2.760252 0.827722 0.547457
H -5.186560 0.912601 0.864985
H -5.196575 -0.691521 1.597992
H -5.022397 -0.006144 -1.369039
H -5.861194 -1.374656 -0.607057

[Na3I2(Br(CH2)3COO)]
E = -3963.595601
C -5.051771 -0.420125 0.744335
C -6.401852 -0.864035 0.178965
C -7.056900 0.469857 -0.189559
O -5.954620 1.378555 -0.454276
C -4.817193 0.916089 0.077103
O -3.785492 1.544030 -0.002333
Na -1.581373 1.136784 -0.056204
H -7.667895 0.437841 -1.088336
H -7.642368 0.893433 0.628570
H -7.003432 -1.434779 0.883861
H -6.255635 -1.474613 -0.712671
H -4.217457 -1.093505 0.550368
H -5.088324 -0.247030 1.824204
Br 0.980177 2.221841 0.020655
Na 3.686621 2.124782 0.029047
I 4.582883 -0.591573 0.016302
Na 1.591314 -0.727430 -0.015700
I -1.171676 -1.799231 -0.083277

[Na4I3(Br(CH2)3COO)]
E = -4423.843840
C 4.747127 0.388817 0.201734
O 3.634377 0.831428 0.057432
O 4.967458 -0.827413 0.718609
C 6.388530 -1.096202 0.832350
C 7.066018 -0.094966 -0.108626
C 6.062531 1.059494 -0.132920
H 6.669727 -0.949605 1.876755
H 6.541328 -2.138230 0.562695
H 8.054928 0.192955 0.243302
H 7.173438 -0.526297 -1.104529
H 5.987338 1.589446 -1.079810
H 6.260481 1.802446 0.644969
Na 1.404155 0.466104 0.146581
Br 0.045047 -0.158260 2.621879
Na -1.950070 1.457990 1.525183
I 0.340041 -1.979045 -1.557801
Na -1.235998 -2.053994 1.021512
I -3.836638 -0.617976 0.313784
Na -1.831048 0.103977 -1.881491
I -0.596256 2.677748 -0.884328

HI
E = -298.399954
H 0.000000 0.000000 -1.585028
I 0.000000 0.000000 0.029906

[(Br(CH2)3COOH)]
E = -2881.313111
C 1.137126 1.347205 -0.170684
C -0.266411 1.712765 0.272848
C -1.391900 1.055608 -0.532934
C -1.949379 -0.198381 0.103330
O -2.463006 -1.040639 -0.821446
Br 1.550089 -0.580102 0.001561
O -2.001133 -0.420555 1.283968
H 1.888932 1.847607 0.432582
H -1.089641 0.837484 -1.557743
H -2.243271 1.740055 -0.603151
H -0.338560 2.800541 0.167857
H -0.403245 1.487471 1.331223
H 1.307534 1.571872 -1.220798
H -2.838363 -1.795092 -0.340147

[Na4I4(Br(CH2)3COOH)]
E = -4722.252892
I 0.079593 -0.942394 2.313325
Na 2.444615 -1.938023 0.657852
I 4.339326 0.435605 0.814749
Na 1.645381 1.594656 1.634780
Na 2.790220 0.803963 -1.794847
I 1.489376 -1.902651 -2.198873
Na -0.753436 -0.250008 -0.797135
I 0.489563 2.588549 -0.963514

Br -5.523787 1.362943 0.318252
O -3.344808 -1.797482 1.524553
C -3.629101 -1.533034 0.257292
O -2.833722 -1.041398 -0.522130
C -5.015072 -1.985799 -0.135342
C -5.683709 -1.133599 -1.218527
C -6.539533 0.006794 -0.700926
H -7.308766 -0.337235 -0.013843
H -7.001941 0.557560 -1.514711
H -6.354705 -1.771969 -1.802776
H -4.926275 -0.760019 -1.908542
H -4.877042 -3.005545 -0.509953
H -5.631367 -2.067254 0.760143
H -2.415642 -1.542894 1.727979

[Na2I2(Br(CH2)3COOH)]
E = -3801.775540
I 3.593631 -2.168103 -0.048768
Na 4.339062 0.617601 0.043276
I 1.986841 2.353316 -0.116337
Na 1.080270 -0.603072 -0.161592
O -1.501098 1.959622 0.546928
C -1.871114 0.692340 0.454760
O -1.097012 -0.220913 0.216535
C -3.350397 0.509721 0.667473
C -3.785307 -0.948863 0.736970
C -5.271937 -1.133801 0.968233
Br -6.371055 -0.487434 -0.546471
H -5.533141 -2.182484 1.070261
H -5.634133 -0.583997 1.833505
H -3.482191 -1.473541 -0.169711
H -3.268366 -1.444266 1.565863
H -3.858534 1.028070 -0.151034
H -3.625108 1.056119 1.573999
H -0.531883 2.058142 0.383331

[(NaI)2((CH2)4COO)]
E = -1266.261713
C -0.328919 2.537056 -0.076664
C -1.713079 3.024971 -0.503628
C -2.772068 1.980607 -0.868791
C -3.490532 1.315413 0.312053
C -2.721158 0.279260 1.107381
I -2.411523 -1.609097 0.014482
H -3.262597 -0.051040 1.988482
H -1.715373 0.585636 1.370276
O 0.440115 3.367121 0.453384
O 0.001261 1.329025 -0.312467
H -2.076942 3.702562 0.272640
H -1.527412 3.662172 -1.374269
H -3.538220 2.480050 -1.467364
H -2.330898 1.217644 -1.513682
H -3.774385 2.092960 1.034661
H -4.428619 0.874043 -0.032316
Na 0.586801 -0.720314 -0.660444
I 3.442506 -0.667151 0.001852
Na 2.198281 1.964324 0.470120

[Na2I(Br(CH2)4COO)]
E = -3542.644237
C -0.929555 2.250516 -0.071017
C -2.388729 2.518522 -0.439931
C -3.264793 1.326185 -0.835854
C -3.842643 0.503256 0.322661
C -2.892907 -0.396789 1.085184
Br -2.306778 -1.985277 0.010782
H -3.364823 -0.853751 1.949534
H -1.965418 0.087303 1.367751
O -0.276511 3.186960 0.436274
O -0.428838 1.106781 -0.325475
H -2.836672 3.085103 0.380581
H -2.341907 3.221447 -1.277494
H -4.114873 1.711321 -1.404890
H -2.713184 0.673630 -1.515330
H -4.263583 1.188743 1.070344
H -4.680931 -0.097845 -0.037418
Na 0.377486 -0.872399 -0.629931
I 3.210226 -0.449798 0.005448
Na 1.661712 2.031306 0.407037

[Na4I3(Br(CH2)4COO)]
E = -4463.124608
C -4.034393 -1.090353 0.300598
Br -4.243594 0.897167 0.307123
Na -1.307644 1.262810 0.052416
C -4.534703 -1.720708 -0.982452
C -3.720644 -1.473206 -2.257829
C -2.352400 -2.164075 -2.310718
C -1.136262 -1.377686 -1.827584
O -0.070929 -2.027344 -1.622448
Na 1.070610 -2.544869 0.200048
I 3.667886 -1.180366 -0.408407
Na 1.200232 0.057901 -1.894255
I 1.190980 2.861556 -0.523939
Na 2.312962 0.834267 1.402493
I -0.189758 -0.705663 2.290778
O -1.201040 -0.123931 -1.718593
H -2.113681 -2.412740 -3.349908
H -2.363719 -3.123564 -1.787562
H -3.590321 -0.401845 -2.419350
H -4.322115 -1.840346 -3.092494
H -4.573208 -2.800928 -0.788526
H -5.568836 -1.408788 -1.145850
H -2.980304 -1.258487 0.503561
H -4.616366 -1.401895 1.162414

Na2I2
E = -920.431318
I -0.000000 -2.378999 -0.000016
Na 1.697220 -0.000000 0.000079
I 0.000000 2.378999 -0.000016
Na -1.697220 0.000000 0.000079

[I(CH2)4COOH]
E = -644.231928
C -0.865560 1.237940 0.773741
I -2.053543 -0.378878 -0.089627
C 0.191966 1.790769 -0.165384
C 1.317180 0.837695 -0.574037
C 2.169694 0.345759 0.607821
C 3.331121 -0.504408 0.157550
O 3.445251 -1.689692 0.326746
O 4.269311 0.227997 -0.494224
H -0.292914 2.173379 -1.066229
H 1.963548 1.355929 -1.284550
H 0.895380 -0.023466 -1.098113
H 2.565740 1.205414 1.155926
H 1.582036 -0.263483 1.292411
H 0.630236 2.661721 0.340404
H -1.603215 1.991058 1.032812
H -0.454887 0.813718 1.685211
H 4.968942 -0.386720 -0.765983

[(NaI)2(Br(CH2)4COOH)]
E = -3841.067859
Br 1.407597 -2.143990 -1.289907
C 2.844289 -0.802011 -1.609512
C 4.014515 -0.956869 -0.654798
C 3.679867 -0.825267 0.832316
C 3.223403 0.587322 1.255903
C 2.292730 0.532242 2.433682
O 2.764416 1.126649 3.534040
O 1.201905 -0.004334 2.414637
H 2.088201 1.051858 4.228127
H 2.644992 1.063839 0.459044
H 4.074852 1.228251 1.478019
H 2.900318 -1.548550 1.075378
H 4.556369 -1.105655 1.419687
H 4.750619 -0.195959 -0.943099
H 4.489959 -1.925514 -0.823829
H 2.330862 0.153516 -1.537473
H 3.140352 -0.974052 -2.639707
Na -0.340472 -0.395546 0.746788
I -3.276051 -0.982729 0.595186
Na -2.788605 1.393178 -0.964332
I -0.004740 2.230408 -0.902948

(CH2)4
E = -157.162099
C 0.068192 1.080339 0.122602
C -1.080479 0.068237 -0.122655
C -0.068250 -1.080290 0.122638
C 1.080561 -0.068315 -0.122639
H -0.123559 -1.952981 -0.528608
H -0.089721 -1.420976 1.159210
H 1.953270 -0.123074 0.528622
H 1.421093 -0.089690 -1.159291
H -1.421128 0.089764 -1.159234
H -1.953119 0.123294 0.528681
H 0.089668 1.420644 1.159320
H 0.123349 1.953197 -0.528379

CO2
E = -188.658232
C 0.000000 0.000000 0.000000
O 0.000000 0.000000 1.159731
O 0.000000 0.000000 -1.159731
